# Redox-Additive Gel Polymer Electrolyte Based on the Biodegradable Polymer Pectin for Supercapacitors with Enhanced Thermal Stability

**DOI:** 10.1021/acsaem.5c01039

**Published:** 2025-06-22

**Authors:** Niyaz Ahmad, Alessia Rinaldi, Michele Setti, Michele Sidoli, Silvio Scaravonati, Vincenzo Vezzoni, Giacomo Magnani, Mauro Riccò, Chiara Milanese, Maria-Magdalena Titirici, Daniele Pontiroli

**Affiliations:** † Nanocarbon Laboratory, cIDEA & Department of Mathematical, Physical and Computer Sciences, 9370 University of Parma, Parco Area delle Scienze 7/A, 43124 Parma, Italy; ‡ GISEL National Centre of Reference for Electrochemical Energy Storage Systems, INSTM National Interuniversity Consortium of Materials Science and Technology, Via Giusti 9, 50121 Firenze, Italy; § Pavia Hydrogen Lab, Chemistry Department, Physical Chemistry Section, University of Pavia, C.S.G.I., I-27100 Pavia, Italy; ∥ Department of Chemical Engineering, 4615 Imperial College London, London SW7 2AZ, U.K.; ⊥ Advanced Institute for Materials Research, Tohoku University, 2 Chome-1-1 Katahira, Aoba Ward, Sendai, Miyagi 980-8577, Japan

**Keywords:** gel polymer electrolyte, biodegradable electrolyte, supercapacitor, biomass waste, activated carbon

## Abstract

The implementation of environmentally green materials in energy storage technologies is essential to ensure a fair and ethical transition to net zero. In this work, we present a gel electrolyte (GPE) based on pectin, a biodegradable natural biopolymer synthesized by using lithium chloride (LiCl) and potassium iodide (KI) as redox additives to enhance the performance of a supercapacitor. GPE shows enhanced thermal stability and flame retardancy, as confirmed by thermogravimetric and differential scanning calorimetry analysis. The optimized redox-additive GPE exhibits high flexibility and outstanding electrochemical properties including a high ionic conductivity (σ = 43 mS cm^–1^) at room temperature and a wide stable potential window (∼2 V vs Ag/Ag^+^). The optimized GPE, with a redox additive and without, was tested with activated carbon electrodes derived from melon peel waste in symmetric supercapacitors. The addition of a redox additive to GPE films directly influences the performance of supercapacitors, leading to a 5 times increase in the specific capacitance (∼437 F g^–1^) and specific gravimetric energy density (∼34 Wh kg^–1^). The optimized supercapacitor exhibits stable cycling performance up to ∼8000 cycles by having an initial ∼31% fade in capacitance and a high Coulombic efficiency close to 99–100%.

## Graphical abstract

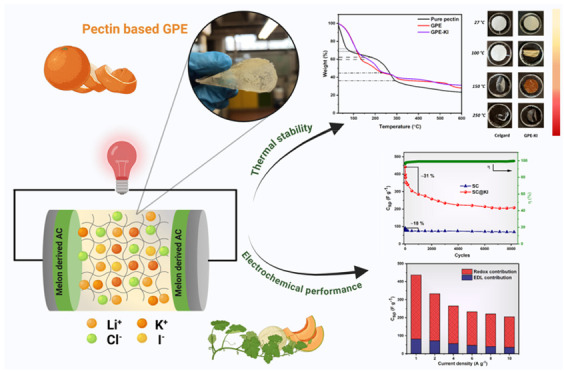

## 1 Introduction

The ongoing research in sustainable and high-performance energy storage materials is the top priority of modern materials research, especially due to the increasing global energy demand and environmental concerns associated with fossil fuels and critical materials. Electric double-layer capacitors (EDLCs), also known as supercapacitors (SC), have emerged as promising candidates for efficient energy storage applications due to their high-power density, fast charge–discharge cycles, cost-effectiveness, and long cycle life.^1–3^ However, their still low energy density as compared to batteries has prompted extensive research into innovative, sustainable materials that can boost their performance.^4^ Ongoing research is primarily focused on improving electrode and electrolyte performance to increase the energy density of SCs while paying attention to sustainability.^5^

Over the decades, the research on the development of SCs mainly focused on the preparation and modification of electrode materials, by tailoring surface area, structure, and porosity.^5^ Different types of carbon, such as porous activated carbon (derived from biomass wastes, polymers, fabrics, fibers, etc.),^6^ graphene, carbon nanofibers (CNFs), carbon nanotubes (CNTs),^7^ carbon aerogels,^8^ etc.,^9^ are employed as electrode materials in SCs.

In addition to the electrode, the electrolyte is an important component in SCs, since the performance of devices is influenced by faster ionic conductivity and a proper interaction between the electrode and the electrolyte. Most of the SCs employ aqueous or organic electrolytes, which have several disadvantages such as corrosion, limited transportability, fast volatility, and flammability.^10^ Aqueous electrolytes offer unique properties, such as high ionic conductivity, low cost, and low toxicity, but they suffer from a low operating voltage window, which leads to a low energy density.^11^ On the other hand, organic electrolytes based on solvents such as ethylene carbonate (EC), adiponitrile (ADN), propylene carbonate (PC), and dimethyl carbonate (DMC) can sustain higher energy densities but suffer from safety and sustainability issues.^12,13^

Polymer-based electrolytes have attracted great attention due to their excellent flexible nature, suitable mechanical and thermal stability, and wide electrochemical voltage window performance.^14–16^ Among these, gel polymer electrolytes (GPEs) are unique, comprising a polymer matrix that has been swollen by a liquid electrolyte.^17–19^ The mechanical stability of solid polymers and the ionic conductivity of liquid electrolytes are combined in this structure. GPEs are distinct from solid polymer electrolytes (SPEs), which frequently have worse conductivity and rely on only ion transport across the polymer chains. GPEs provide better safety and less leakage in comparison to liquid electrolytes. Several studies report how different polymers such as poly­(vinylpyrrolidone) (PVP), poly­(vinyl alcohol) (PVA), PCL (poly-ε-caprolactone), poly­(methyl methacrylate) (PMMA), and poly­(vinylidenefluoride-*co*-hexafluoropropylene) (PVdF-HFP) can be successfully used to design high-performance gel electrolytes.^9,20,21^ In most studies, the polymer or liquid electrolytes used to prepare gel electrolytes are based on synthetic polymers and expensive ionic liquids (such as EMIMBF_4_, BMITFSI,^22,23^ etc.) to maximize the performance of SCs, but they suffer from environmental, cost, and safety issues.

To overcome these shortcomings, recent research has focused on developing safer, more cost-effective, and environmentally friendlier gel electrolytes using natural biopolymers such as chitosan, lignin, cellulose, alginate, etc.^24–27^ Among different biopolymers, pectin has gained huge attention due to its natural abundance, environmental friendliness, and low cost.^28^ Pectin can be found in plant tissues and is plentiful in vegetables and plants.^28^

It has good film-forming properties and mechanical flexibility, making it an appealing matrix for the production of biocompatible composites, edible films, and coating materials.^29^ Commercial pectin is mostly derived from citrus fruit peels (lemon, orange) or from other fruit peels.^30,31^ It is composed of galacturonic acid units linked by α-(1→4)-glycosidic bonds with additional neutral sugars such as rhamnose, arabinose, and galactose.^28,31^ Pectin contains various functional groups, for example –OH, –NH_2_, –CONH_2_, and –COOH, which increase the degree of swelling in polar solvents and allow the fast and easy dissociation of salts, which is crucial for high ionic conductivity.^26,32^

Despite the benefits in different applications, natural biopolymer-based gel electrolytes still have several shortcomings, such as poor interfacial bonding and significant losses in electrochemical performance upon mechanical strain or in terms of long-term stability.^26^ In recent years, incorporation of redox additives into gel electrolytes/liquid electrolytes has been reported to enhance the electrochemical performance of the SCs.^33,34^ It was observed that redox additives can enhance the ionic conductivity of the electrolyte (liquid/gel) while promoting pseudocapacitive reactions that allow fast, reversible faradic reactions at the electrode/electrolyte interface, leading to an increase in the charge storage capacity of the SCs. Across the literature, different alkali halides, for example, KI, LiI, NaI, and neutral hydroquinone (HQ), have been reported to substantially enhance the performance of SCs.^35,36^ To address the shortcomings in biopolymer-based SCs, discussed above, in particular the pectin case, we added the redox additive KI to solve the poor electrochemical performance of SCs. Incorporating this redox additive into the pectin-based gel electrolyte has dual benefits, as it can promote pseudocapacitive reactions while maintaining the environmentally friendly nature.

In this work, we explored the preparation and characterization of the pectin-based gel electrolyte (with and without the redox additive KI) and analyzed its effect on the performance of supercapacitors based on carbon electrodes. The thermal properties of the gel electrolyte films were analyzed by thermogravimetric analysis (TGA) and differential scanning calorimetry (DSC) measurements. Additionally, the flame retardancy and thermal stability of the gel electrolytes were also tested. The electrochemical studies of the gel electrolytes are carried out by measuring the ionic conductivity and electrochemical stability window (ESW). Porous activated carbon (derived from melon waste) based supercapacitors are fabricated with the prepared gel electrolytes (with and without the redox additive KI). A comparative analysis of SCs has been performed by means of electrochemical impedance spectroscopy (EIS), cyclic voltammetry (CV), and galvanostatic charge–discharge (GCD) measurements. The advantage of KI as a redox additive on gel electrolyte films has been observed directly in terms of specific capacitance, energy, and power density.

## 2 Experimental Section

### 2.1 Preparation of the GPE films

The redox-active GPE and GPE with no redox species were prepared through a solution casting technique (Figure 1). First, the proper ratio of natural polymer pectin (from citrus peels, Merck KGaA, Germany) and LiCl (≥99%, Merck KGaA, Germany) was optimized by varying the amount of pectin and LiCl. Different amounts of pectin, namely, 30, 40, and 50 (wt %), were mixed together with 70, 60, and 50 (wt %) of LiCl, respectively, to obtain a total amount of 1 g. The mixture was then added to 20 mL of deionized water and continuously stirred on a magnetic stirrer for 12 h. The gel electrolyte films with a redox additive were obtained by adding 0.1, 0.2, 0.3, and 0.4 g of KI (99%, Merck KGaA, Germany) into the LiCl–pectin solution and stirring again for another 12 h. Then, the solutions were poured into glass Petri dishes and dried at 45 °C overnight. After that, samples underwent further drying at 60 °C for 4 h to obtain a gel. After complete solvent evaporation, a free-standing and mechanically stable film was obtained (Figure 1, top right). The thickness of the obtained films was in the range of 350–400 μm, and the prepared gel electrolyte films were stored in a dry atmosphere to avoid moisture/contamination.

**Figure 1:**
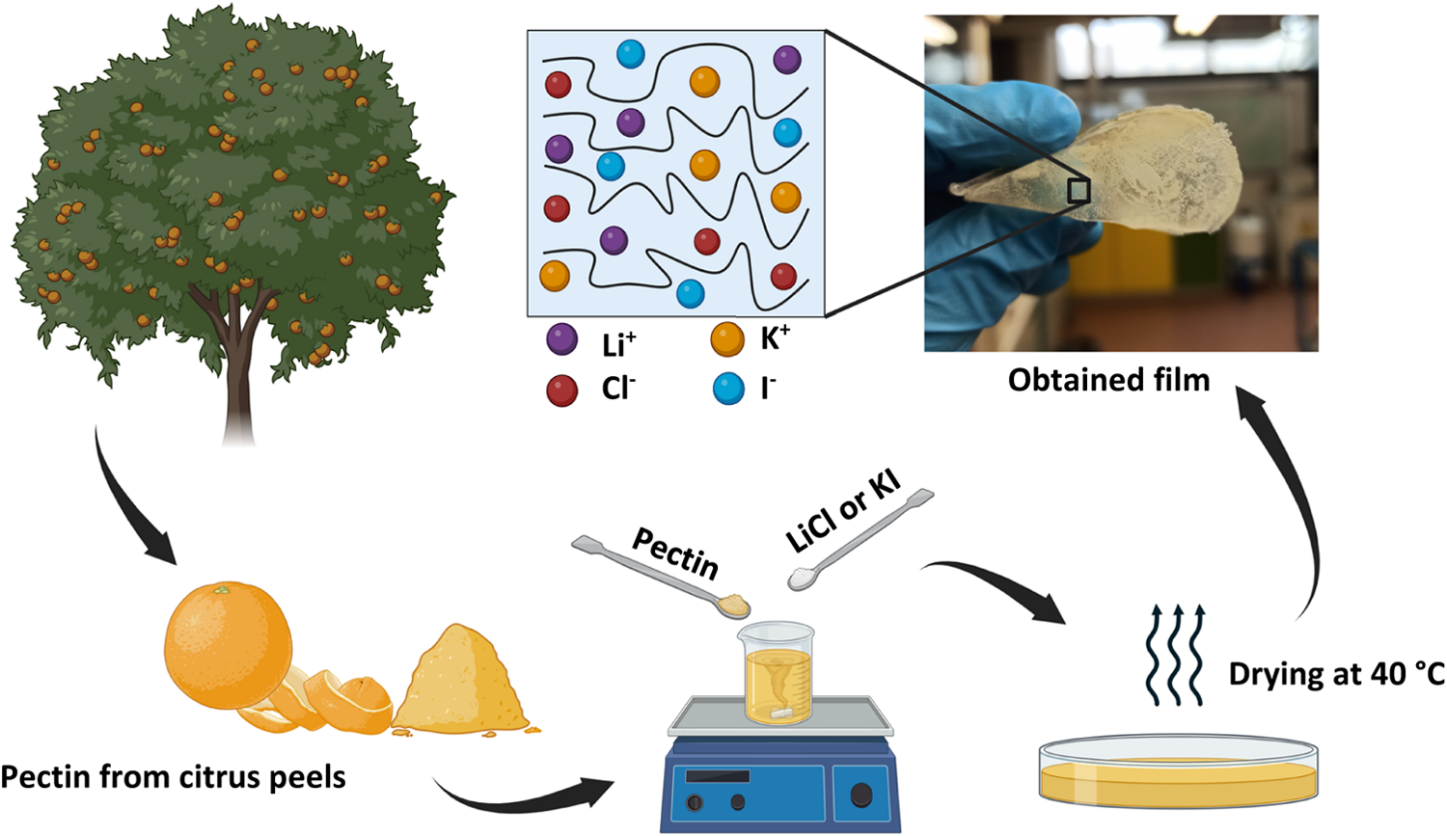
Progressive preparation steps of the GPE films.

The 50 (wt %) pectin and 50 (wt %) LiCl ratio was chosen, thanks to the superior mechanical stability of the free-standing film. Pictures of the membranes are shown in Figure S1. The amount of the redox additive (KI) in pectin/LiCl electrolyte has been optimized based on ionic conductivity measurements. The gel electrolyte films with 0.1 g (9 wt %) of KI have been chosen, due to their highest ionic conductivity. The redox-additive gel electrolyte film is referred to as “GPE-KI”.

### 2.2 Preparation of the Electrodes

Hierarchical porous activated carbons (ACs) derived from melon waste have been used as high-performance carbon electrodes to test the gel electrolyte films in symmetric supercapacitors.^37^ The pyrolyzed melon waste was mixed with KOH (ACEF S.p.a., Italy) in a 1:6 (wt %) ratio and carbonized at 800 °C under an inert atmosphere. The detailed information on the preparation of ACs has been described in our previous work.^1,2^ The prepared AC exhibits a total BET specific surface area of 3035 m^2^/g with a micropore volume of 1.42 cm^3^/g, which is excellent for high-performance supercapacitors. The carbon electrodes were prepared via drop casting: a slurry was prepared by mixing AC powder, carbon black (Super C 65, Timcal, Switzerland), and PVdF-HFP (pellets, Merck KGaA, Germany) binder in a ratio of 80:10:10 (wt %) in a mortar and ground adding acetone to obtain a homogeneous mixture. Then, the slurry was drop-cast on a circular graphite sheet (diameter of 16 mm) and vacuum-dried at 90 °C overnight. The total mass of the electrode materials, including binder and carbon black, was around 0.8–1.1 mg cm^–2^.

### 2.3 Fabrication and Assembly of Supercapacitors

The quasi-solid-state supercapacitor cells were assembled by sandwiching gel electrolyte films between two symmetric carbon electrodes. The gel electrolyte films were dried at 40 °C for 2 h before assembly of the supercapacitor cells to remove moisture. The prepared supercapacitors were assembled in coin cells (CR-2032). Two types of supercapacitor cells were assembled, with GPE and with GPE-KI, and will be referenced as SC and SC@KI, respectively. The configuration of the cells is given as follows

SC:AC|GPE|AC

SC@SKI:AC|GPE−KI|AC

where GPE: 50 (wt %) pectin +50 (wt %) LiCl, and GPE-KI: 45.5 (wt %) pectin +45.5 (wt %) LiCl + 9 (wt %) KI.

Cork-LIG-based supercapacitors were made by dropping liquid solutions of GPE-KI on top of the printed electrodes. After that, they were dried at 60 °C in an oven, and then silver paste was used to guarantee appropriate contact with the potentiostat. To prevent the electrolyte from absorbing water from the ambient, the devices were enclosed in a home-built portable chamber filled with argon gas and supplied with measurement cable connections.

### 2.4 Characterization Techniques

Thermal gravimetric analysis (TGA) on pectin-based electrolytes was conducted using a PerkinElmer TGA 8000 (PerkinElmer, Norwalk, CT) thermogravimetric analyzer, equipped with a Pt crucible (sample mass of approximately 10 mg) at a heating rate of 10 °C/min in a temperature range of 25–600 °C. All of the measurements were run under a constant flux of dry nitrogen (30 mL/min). Differential scanning calorimetry (DSC) evaluation was carried out with a PerkinElmer DSC 6000. The measurement was performed at atmospheric pressure under a constant flow of nitrogen (20 mL min^–1^) in the temperature range from −50 to 300 °C. The enthalpy of the endothermic and exothermic events is determined using Pyris software.

The morphology of the gel electrolyte films was analyzed using a Hitachi TM4000Plus II tabletop scanning electron microscope (SEM). The small pieces of the films were fixed on carbon tape and fixed to the sample holders. The SEM operated at an accelerating voltage of 15 kV, and both secondary electrons (SE) and backscattered electrons (BSE) were utilized to achieve a detailed and accurate visualization of the surface structure of the samples.

A 3Flex analyzer (Micromeritics, Norcross, Georgia) was used to measure the specific surface area of the AC. Samples were heated for 4 h in a nitrogen flow at 120 °C using a FlowPrep system (Micromeritics, Norcross, Georgia) before measurement. The 3Flex ports were vacuum-degassed at 120 °C for 2 h to remove surface contaminants and moisture. The specific surface area of AC was calculated using the Brunauer–Emmett–Teller (BET) technique, which ensured that the Rouquerol criterion for reliable measurements was satisfied.

#### 2.4.1 Electrochemical Characterization

A 1010E Interface Potentiostat (Gamry Instruments, PA) was used to measure linear sweep voltammetry (LSV), EIS, and CV. Analyses were performed on both the as-prepared electrolyte and the fully built EDLC devices. LSV measurements were performed using a two-electrode configuration with a constant scan rate of 10 mV/s to determine the GPE’s operational voltage window. EIS measurements were performed in a frequency range from 10 mHz to 10^5^ Hz using a 10 mV AC signal. CV testing was performed on the final EDLCs. Initially, CV scans were carried out in the voltage range from 0 to 1.5 V at a constant scan rate of 10 mV/s to investigate the operational voltage window of the full device. Then, the optimal voltage range was chosen, and CV scans at different scan rates from 10 to 100 mV/s were performed.

Galvanostatic charge/discharge (GCD) analysis was performed to determine the capacitance, efficiency, and capacitance retention of SC and SC@KI supercapacitors. GCD measurements were performed with the BTS-4008 (5V50 mA, Neware, Hong Kong) battery tester equipment. The measurements were carried out using both constant current (chronoamperometry) and constant voltage modes.

To identify the ideal working voltage range, the EDLCs were charged and discharged at voltages ranging from 0 to 2.5 V while maintaining a constant current of 1 A/g. The EDLCs were then charged and discharged at different current densities in the optimal voltage range. Furthermore, the EDLCs were charged and discharged 8000 times with a constant current density of 1 A/g.

## 3 Results and Discussion

### 3.1 Characterization of Gel Electrolyte Films

The electrochemical properties of the gel electrolyte films have been analyzed mainly by performing ionic conductivity and electrochemical stability window (ESW) experiments. The ionic conductivity of the films was measured by recording EIS spectra in the cell configuration: SS| gel electrolyte films | SS, where SS is a stainless steel disk. The bulk resistance *R*_b_ of each film was calculated from the EIS spectra, and the ionic conductivity (σ) was calculated from the following equation

1 $$\sigma =\frac{L}{\left(R_{b}\times A\right)}$$

where *L* and *A* are the thickness and contact area of the electrolyte films, respectively.^38^ The ionic conductivity of the GPE (without KI/No KI) has been calculated to be ∼4.1 × 10^–3^ S cm^–1^ at room temperature. The ionic conductivity variation with respect to the amount of the redox additive (KI) is reported in Figure 2A. Initially, the addition of a small amount (9%) of KI was followed by an increase in ionic conductivity. Any subsequent addition of KI was followed by a gradual decrease in ionic conductivity. The initial increase in ionic conductivity is related to an increased concentration of charge carriers (K^+^ and I^–^), while the gradual decrease after the maximum is probably due to the ion pairing and/or aggregation among the different cations and anions (Li^+^, Cl^–^, K^+^ and I^–^), which reduces the number of free charge carriers. The optimized ionic conductivity of the redox-additive gel electrolyte film was found to be ∼4.3 × 10^–2^ S cm^–1^ at room temperature.

**Figure 2:**
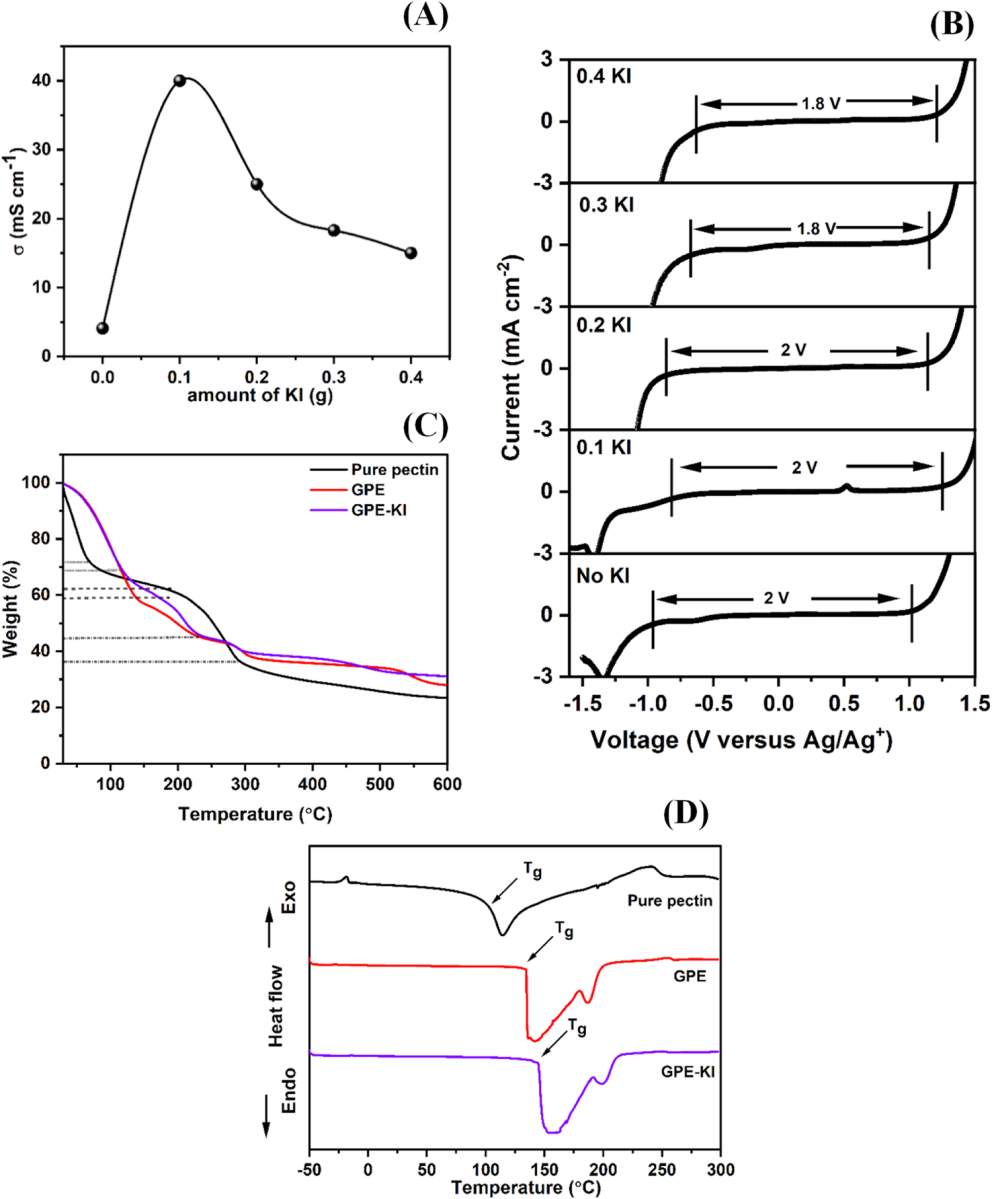
(A) Variation of ionic conductivity of the gel polymer electrolyte film with redox additive (KI) content in the pectin/LiCl composition, (B) electrochemical voltage window of the GPE without KI (No KI) and gel polymer electrolyte films with different amounts of KI content, (C) TGA and (D) DSC curves of the pectin film (pure), GPE (without KI), and GPE-KI (with 0.1 g of KI).

Figure 2B shows the ESW of all gel electrolyte films measured by performing LSV, obtained at a 5 mV s^–1^ scan rate on a cell configuration: SS| gel electrolyte films | Ag, where SS is a stainless steel disk, which acts as a working electrode while Ag is a silver disk acting as a reference/counter electrode. All of the gel electrolyte films exhibit voltage stability in the voltage range from 1.8 to 2 V versus Ag/Ag^+^. Moreover, the GPE (without redox additive/NO KI) and gel electrolyte with 0.1 g of KI (GPE-KI) show a stable voltage window of ∼2 V versus Ag/Ag^+^. The obtained voltage window is suitable for the application of high-energy-density electrochemical supercapacitors.

The thermal stabilities of the optimized gel electrolyte films GPE (without redox additive) and GPE-KI (with redox additive) have been investigated from TGA and DSC analysis, and the results have been compared with pure pectin film. Figure 2C shows the TGA curves of the pure pectin film, GPE, and GPE-KI. All three films show an initial weight loss below 60–100 °C due to water/moisture evaporation and low-boiling-point volatile components. The pure pectin film shows thermal decomposition in the temperature range of 200–300 °C, while both GPE and GPE-KI show lower decomposition temperatures in the range of 150–250 °C, which indicates that LiCl (and KI in the GPE-KI film) lowers the thermal decomposition temperature, possibly due to the catalytic effects of the salts in the gel electrolyte films. However, the combination of both LiCl and KI in the GPE-KI film enhances thermal stability, as evidenced by the highest residual weight (25–35%) at ∼600 °C.

The thermal properties of the electrolyte films have also been studied through the DSC technique. The recorded DSC curves of pure pectin, GPE, and GPE-KI are reported in Figure 2D. The glass transition temperature (*T*_g_) of pure pectin has been observed at ∼90–94 °C, in agreement with the literature.^39^ Additionally, a sharp endothermic peak at ∼120 °C, related to the loss of water and to the initial breakdown of the saccharide structure, was observed.

In the case of electrolyte films with and without a redox additive (GPE and GPE-KI), higher *T*_g_ values, in the range of ∼130–140 °C, have been observed, which is due to changes in the chain mobility after the addition of the salts. The presence of LiCl/KI reduces the amorphous nature of the electrolyte films, which become more crystalline due to ion aggregation, suggesting the interaction of the polymer chains with the salts.^40^ Moreover, GPE-KI is characterized by the broadest and most shifted to high-temperature (162–165 °C) endothermic peak, as can be observed in Figure 2D. This is due to the enhanced melting/decomposition of crystalline regions formed by the interaction of pectin chains with LiCl and KI. The strong ionic interactions between the additional salts (LiCl and KI) and the functional groups of pectin, specifically the carboxyl and hydroxyl groups, are responsible for the increase in *Tg* shown in our GPE and GPE-KI systems.

The morphology of the gel electrolyte films has also been recorded and is represented in Figure S2. The pure pectin film exhibits a dense and uniform texture with no visible pores or roughness, as can be observed in Figure S2A, while a rough surface and microcracks can be observed in the gel electrolyte film without KI (Figure S2B) after addition of LiCl, which is most likely due to the interaction between the polymer and salt, resulting in partial phase separation or the development of ionic domains. From the cross-sectional part of the film, a rougher and uneven structure can be observed with improved porosity, as visible in Figure S2C, which possibly creates pathways for ionic conduction. Figure S2D,E represents the surface texture and cross-sectional view, respectively, of the optimized redox-additive gel electrolyte film. Both images represent an enhancement in the roughness and porosity in the film with visible microstructures after addition of KI, which significantly improves the ionic conduction and enables redox activity. The enhanced irregular morphology with a more open structure facilitates ion transport in the film. Figure S2F shows the surface morphology of the carbon electrode (activated carbon, carbon black, and a binder). The structure is highly porous and irregular, with interconnected particle networks that enhance surface area. Figure S2G,H shows a clear and continuous contact between the electrode and the gel polymer electrolyte film.^41^ There is no obvious delamination or fracture at the contact, suggesting robust interfacial adhesion. Below the electrolyte, the stratified microstructure of the electrode material is visible; there are no visible spaces or gaps, indicating effective electrolyte penetration and contact.

High thermal stability is important for separators/gel electrolyte films to maintain their shape in case of high temperature, which may be caused by short circuit due to overcharging (thermal runaway).^42^ Figure 3A shows that GPE-KI is more thermally stable than Celgard 2320. Although at high temperatures, shrinking is observed for both materials, Celgard exhibits more severe deformation starting at lower temperatures (150 °C), while deformation in GPE-KI is observed only at higher temperatures (250 °C). This implies that GPE-KI is characterized by better thermal stability and structural integrity than Celgard. This also indicates that in applications involving higher temperatures, GPE-KI is better suited than Celgard.

**Figure 3:**
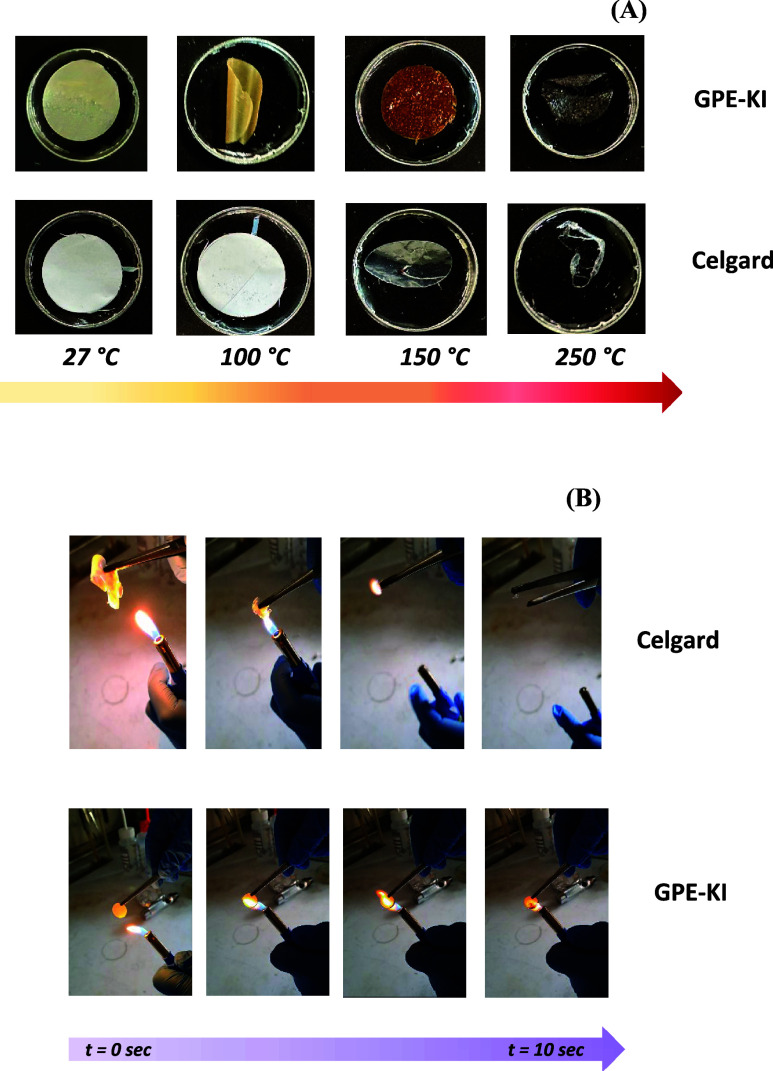
(A) Images of GPE-KI and Celgard after heating at different temperatures for 2 h. (B) Ignition test of Celgard and GPE-KI.

Flame retardancy is also an important factor in ensuring a significant level of safety. As can be seen in Figure 3B, Celgard lights up and burns completely few seconds after exposure to a flame, which demonstrates very poor fire resistance. On the contrary, the gel electrolyte film GPE-KI shows remarkable flame retardancy, partly retaining its structure after exposure to a flame. This implies that GPE-KI can successfully lower the chance of the ignition of the final device.

### 3.2 Performance Study of Supercapacitors/EDLCs

#### 3.2.1 Electrochemical Impedance Spectroscopy (EIS)

The interfacial and bulk kinetics of the supercapacitors have been studied by EIS measurements. The EIS plots of supercapacitors SC and SC@KI have been measured in the frequency range from 0.01 Hz to 100 kHz, and the respective spectra are reported in Figure 4A,B. From these Nyquist plots, it can be noticed that as compared to the SC cell, the SC@KI cell has a steeper approach to the imaginary axis, which represents better ion mobility and lower diffusion impedance.^43^ This highlights the advantage of the redox additive in significantly enhancing the ionic conductivity of the gel electrolyte film.

**Figure 4:**
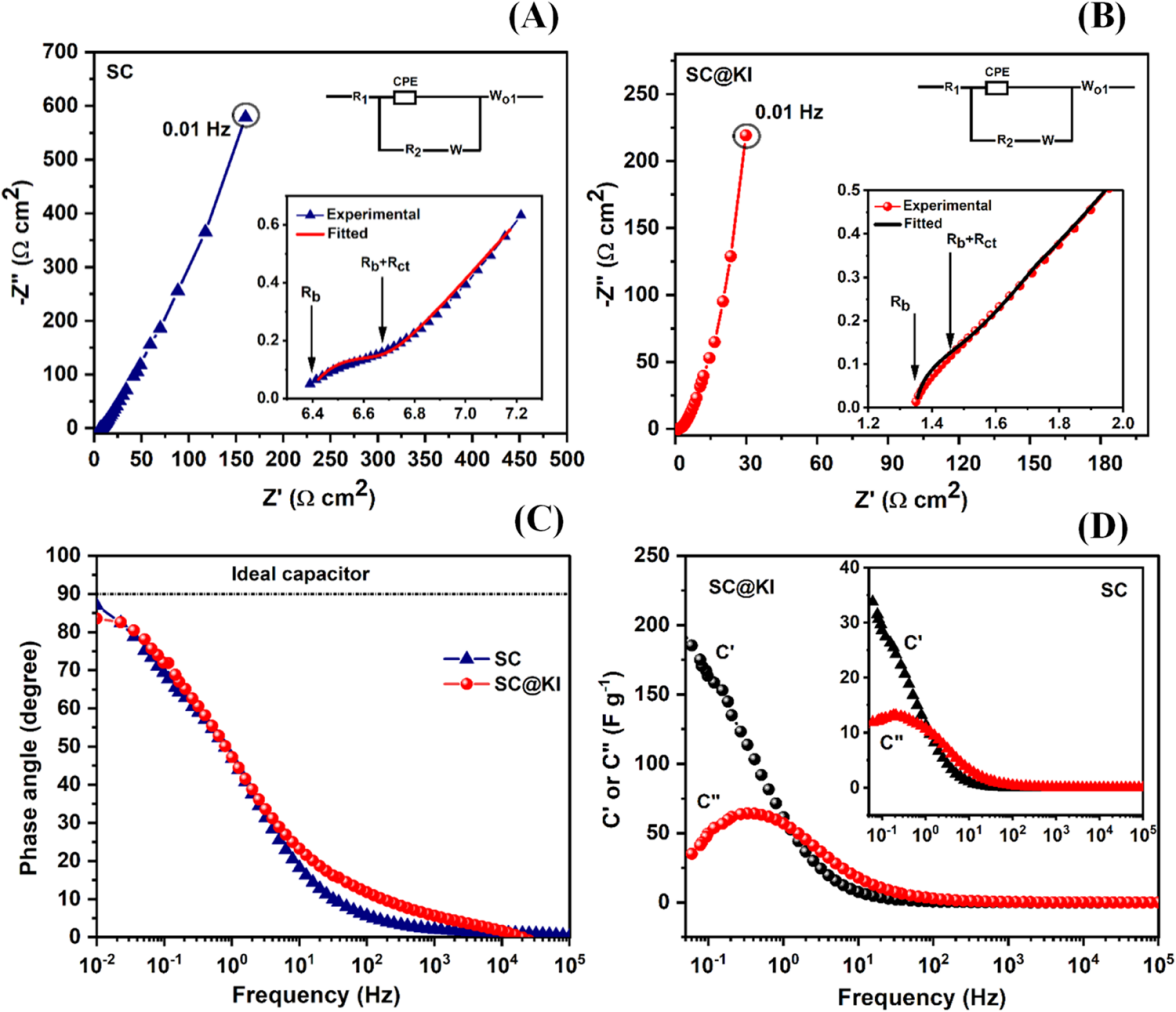
EIS (Nyquist) plots of supercapacitor cells (A) SC, and (B) SC@KI in the frequency range from 100 kHz to 10 mHz (expanded EIS plot in high frequency region and equivalent circuit are also shown in each figure), (C) phase angle versus frequency plot, and (D) real and imaginary values of capacitance (C′ and C″) versus frequency plot of supercapacitor cells (SC@KI and in the inset SC).

Moreover, the insets of Figure 4A,B show expanded plots in the mid-to-high frequency region, which denotes the interfacial and bulk properties of the supercapacitors. The values of bulk resistance (*R*_b_) and charge-transfer resistance (*R*_ct_) have been calculated from the intercepts of semicircular arcs with the *Z*′-axis, as shown by the arrows in the insets of Figure 4A,B, and the obtained values have been listed in Table 1. Additionally, the total impedance (*R*), estimated at 0.01 Hz, has also been listed in Table 1 for both supercapacitors (GPE and GPE-KI). The SC@KI cell shows an almost 5 times lower *R*_b_ value as compared to the SC cell, due to the improved ionic conductivity of the redox-additive gel electrolyte film and to the smooth ion movements through the pores of the carbon electrodes. Moreover, an almost 4 times lower *R*_ct_ value has been observed in the SC@KI cell, which is mainly due to the faster redox reactions and improved electron/ion transport across the electrolyte–electrode interface.^44,45^ Furthermore, the SC@KI cell shows a very low R value compared to the SC cell. This implies that the addition of KI (enhanced pseudocapacitive effect) not only improves the charge transfer at the electrolyte–electrode interface but also increases the overall performance of the supercapacitor (SC@KI) by reducing the energy loss at low frequencies.

**Table 1:** Electrical Parameters were Evaluated from EIS Studies for Both Supercapacitors

EDLCs	*R*_b_ (Ω cm^2^)	*R*_ct_ (Ω cm^2^)	*R* (Ω cm^2^)	*C*_sp_ (F g^–1^)
SC	6.2 ± 0.1	0.35 ± 0.05	156 ± 2	57 ± 4
SC@KI	1.3 ± 0.05	0.125 ± 0.025	31.5 ± 1.5	209 ± 3

The accuracy of the electrical parameters has been analyzed by fitting the Nyquist curve in the mid-to-high frequency region for both cells (SC and SC@KI), as shown in the insets of Figure 4A,B. Different parameters are used to get the best fit of the curves, namely, resistive parameters (*R*_1_ and *R*_2_), Warburg components (*W* and *W*_01_), and constant phase element (CPE), as listed in Table S3. The accuracy of the fitting can be observed, as the values of fitted parameters *R*_1_ and *R_2_* are found to be close to the obtained experimental values *R*_b_ and *R*_ct_ (bulk and charge-transfer resistances), respectively. The detailed information about the fitting of Nyquist curves in the mid-to-high frequency region can be found in our previous work.^45^

The specific capacitance (*C*_sp_) values for both cells (SC and SC@KI) have been calculated from EIS data by using the following expression

2 $$C_{\mathrm{sp}}=\frac{2}{2\pi f\times m\times \left|Z^{\prime \prime }\right|}$$

where (|*Z*″|) is the magnitude of the imaginary component of the impedance, which has been calculated at a frequency *f* of 0.01 Hz and (m) is the mass of the AC material deposited on the single electrode.^45,46^ The *C*_sp_ values listed in Table 1 show the effect of the redox additive in the gel electrolyte film, since it increases the specific capacitance by almost 4 times. The actual values of specific capacitance and energy have been calculated from the GCD analysis, discussed later.

The phase angle versus frequency plots, i.e., bode plots of both cells (SC and SC@KI) are shown in Figure 4C. Both cells show a sharp decrease in the phase angle in the frequency region from 0.01 to 10 Hz, which indicates the dominance of the capacitive nature of cells in a low-frequency zone. Additionally, the SC cell (without the redox additive) shows a slightly better phase angle response than the SC@KI cell, since it is closer to the ideal capacitor phase angle response, i.e., 90 ° (shown by the dotted line in Figure 4C). It may be noted that the phase angle values for SC and SC@KI are ∼87.5 and 83°, respectively. This slight decrease in the phase angle at lower frequencies implies the increased contribution of faradic reactions (due to the redox additive KI), which are slower than the electrostatic charge storage mechanism.

Furthermore, the ion diffusion process at the electrode–electrolyte interface has been analyzed from the real and imaginary capacitance (*C*′ and *C*″) plot against frequency (0.01–100 kHz) for the cell with the redox additive (SC@KI), as shown in Figure 4D. For the SC cell (without the redox additive), the plot is shown in the inset of Figure 4D. The values of *C*′ and *C*″ have been calculated from the real and imaginary impedances (*Z*′ and *Z*″) by using the following expressions.^45,47^

3 $$C^{\prime }\left(\omega \right)=\frac{{-Z}^{\prime \prime }\left(\omega \right)}{\omega {\left|Z\left(\omega \right)\right|}^{2}}$$

4 $$C^{\prime \prime }\left(\omega \right)=\frac{Z^{\prime }\left(\omega \right)}{\omega {\left|Z\left(\omega \right)\right|}^{2}}$$

Both cells exhibit a sharp decrease in *C*′ below ∼5 Hz and a peak in *C*″ against frequency plots. The peak in the *C*″ versus frequency plots denotes a relaxation of ions through the pores of the electrodes. It may be noted that this peak is observed in the low frequency region at ∼2 Hz and represents the transition from resistive to capacitive behavior in cells as frequency decreases.^1,10^ This is typical of porous activated carbon-based supercapacitors.^10,46,48^ Moreover, the peaks of C″ for SC and SC@KI have been observed at ∼0.2 and ∼0.4 Hz, respectively. This clearly shows that redox additive-based cell (SC@KI) is more capacitive in a wider frequency range (transition from resistive to capacitive at a higher frequency) than SC, which is due to the enhanced faradic reactions at the electrode–electrolyte interface.

#### 3.2.2 Cyclic Voltammetry

The electrochemical behavior of supercapacitor cells SC and SC@KI has been characterized by CV analysis in symmetric two-electrode systems. First, the maximum working voltage window of the supercapacitors was optimized by performing CV measurements at varying voltage ranges from 0 to 1.5 V at a constant scan rate of 10 mV s^–1^. An almost rectangular box-like shape (rectangular shape for ideal capacitors) has been observed up to 1.5 V for both cells, as shown in Figures 5A and S4 for cells SC@KI and SC, respectively. The maximum potential stability of cells is assessed as 1.5 V, which is used as the maximum voltage for the rest of the study.

**Figure 5:**
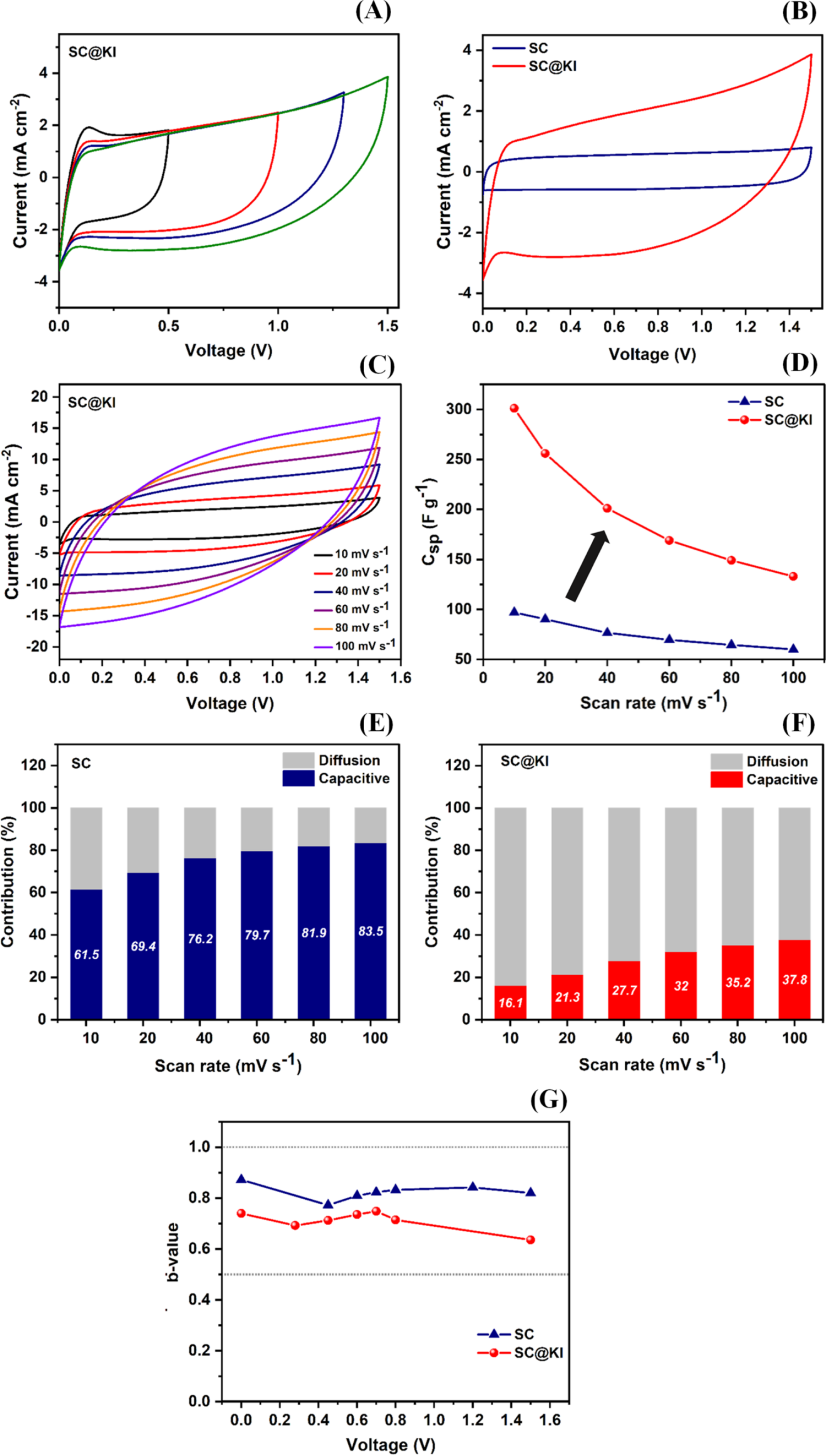
(A) CV curves of the supercapacitor cell SC@KI with varying voltage ranges recorded at a scan rate of 10 mV s^–1^, (B) comparative CV curves of cells SC and SC@KI in the optimized voltage range of 0–1.5 V at a scan rate of 10 mV s^–1^, (C) CV curves of cell SC@KI obtained at different scan rates, (D) specific capacitance (*C*_sp_) versus scan rate plot for both cells, (E and F) capacitive and diffusive contributions calculated from CV curves at different scan rates for cells SC and SC@KI, respectively, and (G) the b-values of cells SC and SC@KI calculated at different potentials.

Figure 5B shows comparative CV curves of SC and SC@KI at a scan rate of 10 mV s^–1^ in the optimum voltage range of 0–1.5 V. SC shows an almost ideal rectangular pattern, as expected from carbon-based supercapacitors due to the purely electrostatic charge storage mechanism. On the other hand, the CV pattern of the SC@KI cell is characterized by an enlarged area due to the presence of the redox reaction occurring at the interface by iodide anions after introduction of the redox additive (KI) in the gel electrolytes,^10^ implying a higher capacitance. The following possible redox reactions can occur at the electrolyte–electrode interface after ionization of the redox additive (KI).^10,11,48^

5 $$\begin{array}{l} 3I^{-}↔I_{3}^{-}+2e^{-}\\ \begin{array}{l} 2I^{-}↔I_{2}+2e^{-}\\ \begin{array}{l} 2I_{3}^{-}↔3I_{2}+2e^{-}\\ I_{2}+6H_{2}O↔2{IO}_{3}^{-}+12H^{+}+10e^{-} \end{array} \end{array} \end{array}$$

These possible multiple redox reactions are due to pseudocapacitive effects and lead to the enhancement of capacitance in the SC@KI cell. The presence of pseudocapacitance is suggested by the absence of well-defined redox peaks in the CV curves. The absence of well-defined redox peaks in the CV curves of SC@KI is due to the quasi-reversible and highly diffusive nature of the iodide–triiodide redox couple within the gel polymer matrix. Additionally, the broad and overlapping features are a consequence of ion transport limitations and the formation of a distributed redox environment within the gel polymer structure. These characteristics hinder the appearance of sharp, well-resolved redox peaks.

The rate capability of cells (SC and SC@KI) has been analyzed by recording CV measurements at different scan rates from 10 to 100 mV s^–1^, as shown in Figures 5C and S5 for the SC@KI and SC cells, respectively. Both cells are capable of maintaining the ideal rectangular shape of the CV curve up to 100 mV s^–1^, showing moderate rate capabilities. Specific capacitance (*C*_sp_) values have been calculated from CV curves at different scan rates for both cells, as shown in Figure 5D, by the following expression^11,45,46^

6 $$C_{\mathrm{sp}}=\frac{\int j\,\;dV}{s\times m\times \Delta V}$$

where ∫*j* d*V* is the area under the CV Curve, s is the scan rate, ΔV is the optimized potential window, and m is the mass of the active material employed on a single electrode. The redox additive-based cell (SC@KI) shows higher capacitance (almost 3 times) than the SC cell at lower scan rates. However, a faster decrease in *C*_sp_ values at higher scan rates can be observed for the SC@KI cell, due to the inherent lower rate capability of the redox contribution. Despite the decrease at a high scan rate, the *C*_sp_ of SC@KI is still quite higher than SC, with almost double the specific capacitance at 100 mV s^–1^. This shows the higher rate capability of the SC@KI cell as compared to that of the SC cell.

The cells were also analyzed by comparing the surface-controlled and diffusion-controlled capacitance contributions, as shown in Figure 5E,F, by using the following expression^49^

7 $$j=k_{1}v+k_{2}v^{1/2}$$

where *k*_1_ and *k*_2_ are constants, (*k*_1_ *v*) denotes the surface-controlled capacitance contribution, and (*k*_2_ *v* ^1/2^) represents the diffusion-controlled capacitance contribution.^50^ The SC cell shows dominant electrostatic capacitive character for all scan rates, as shown in Figure 5E. On the contrary, the SC@KI cell shows a dominant diffusion contribution for all scan rates, as shown in Figure 5F, which is due to the enhanced redox reactions at the interface. This proves that introducing the redox additive KI in the gel electrolyte film leads to pseudocapacitive effects and enables ions to diffuse deeper into the pores of the electrodes. At higher scan rates, the ions have a shorter time to diffuse into the pores of the bulk electrode, which leads to an increase in the fraction of capacitive contribution, but with still a clear dominance of the redox character. This clarifies the role of the redox additive KI to achieve higher capacitance.

Furthermore, an empirical relationship between current (*i*) and scan rate (*v*) was used to understand more about the charge storage mechanism in the cells.^50^ The expression *i = av* ^*b*^ has been used, where *a* and *b* are constants, while *v* is the scan rate.^49^ It may be noted that when the value of *b* is 0.5, it represents an ideal diffusion-type behavior, while if it is 1, it shows an ideal capacitive-type charge storage mechanism.^50^ From Figure 5G, it can be seen that the SC cell shows higher *b* values close to 1, while the SC@KI cell shows lower b values close to 0.5, which is due to the presence of the redox additive KI. This again indicates the more dominant contribution of the diffusion-controlled charge storage mechanism.

#### 3.2.3 Galvanostatic Charge–Discharge

The electrochemical performance and crucial parameters of the supercapacitors (SC and SC@KI) have also been analyzed through GCD measurements. First, the maximum operating voltage window of the cells (SC and SC@KI) has been optimized. Both cells were charged–discharged in the voltage range of 0–1.5 V at a constant current density of 1 A g^–1^, as shown in Figures 6A and S6 for SC@KI and SC cells, respectively. The SC cell shows almost ideal triangular charge–discharge curves up to 1.5 V (can be seen in Figure S6), while SC@KI exhibits a nonlinear charge–discharge curve due to pseudocapacitive reactions along with a double-layer charge storage mechanism. In this voltage range, both cells are able to hold a maximum Coulombic efficiency. Beyond this voltage range, the Coulombic efficiency of the cells starts decreasing below 90%. Hence, 0–1.5 V has been chosen as an optimized voltage range for the cells, and the rest of the analysis was performed in this range. This is also in agreement with the voltage range identified from the CV analysis.

**Figure 6:**
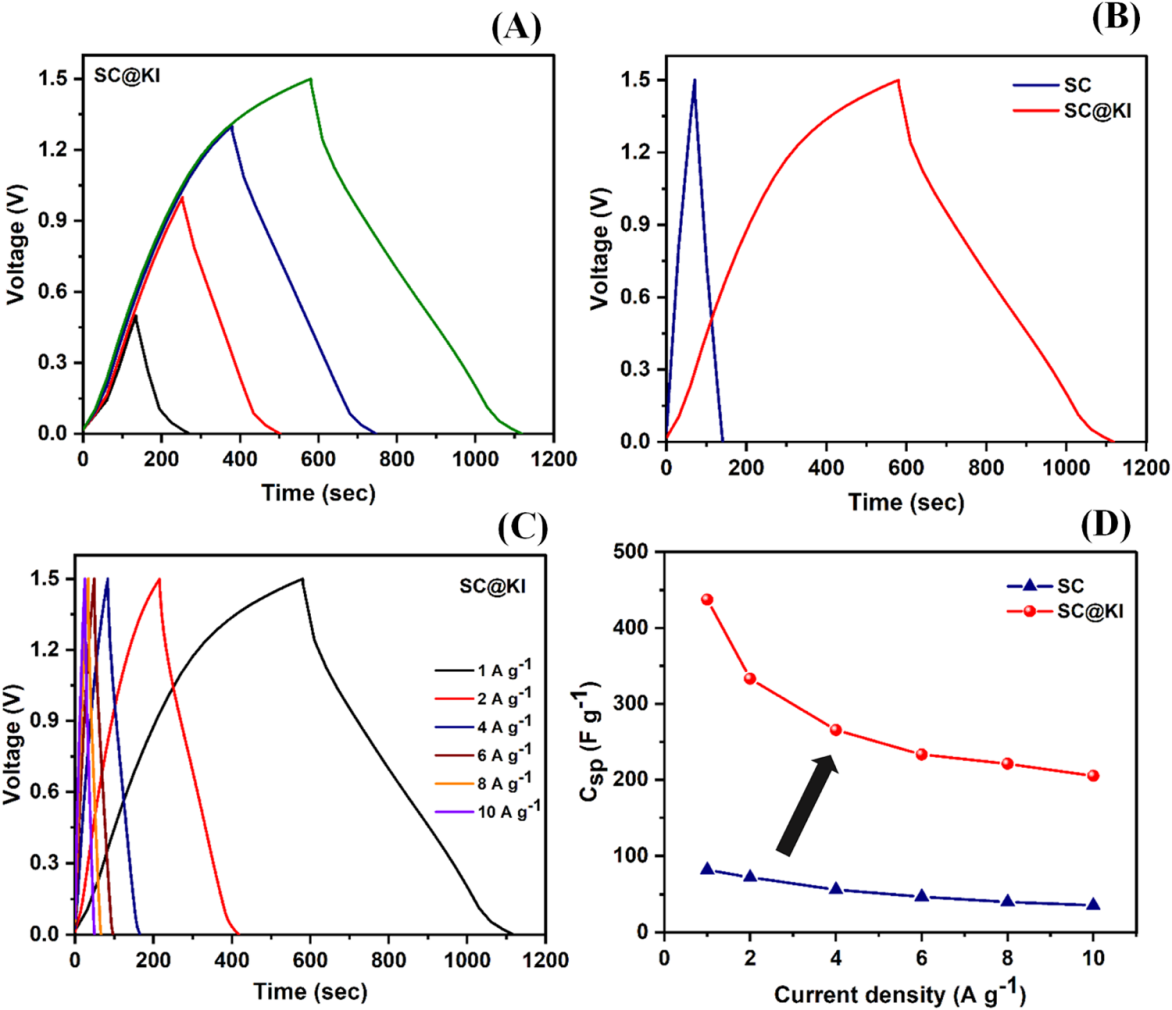
(A) GCD curves of the SC@KI cell with varying voltage ranges recorded at a current density of 1 A/g, (B) comparative GCD curves for cell SC and SC@KI in the optimum voltage range of 0–1.5 V, (C) GCD curves obtained at different current densities from 1 to 10 A g^–1^ for the SC@KI cell, and (D) variation of specific capacitance (*C*_sp_) with respect to current density for both cells.

In Figure 6B, the charge–discharge curves of SC and SC@KI cells have been reported in the chosen optimum voltage window of 0–1.5 V recorded at 1 A g^–1^. The SC (without redox additive) cell shows linear charge–discharge curves, indicating an excellent electrostatic charge storage mechanism at the interface, generally observed in carbon-based supercapacitors.^11,45^ On the contrary, the SC@KI cell shows a nonlinear charge–discharge profile due to the occurrence of faradic reactions, already discussed in the Cyclic Voltammetry section, along with the electrostatic charge storage mechanism at the electrolyte–electrode interface. This results in a longer charge–discharge time, which leads to higher specific capacitance than the SC cell. This nonlinearity could potentially lead to a minor reduction in Coulombic efficiency under prolonged cycling. However, within the tested operational range, these effects appear limited and do not significantly compromise the overall device performance or stability. The values of the specific capacitance (*C*_sp_) for both SC and SC@KI cells have been calculated from the following expression^10,48^

8 $$C_{\mathrm{sp}}=\frac{4j\int V\,dt}{{m\times V^{2}|}_{V_{i}}^{V_{f}}}$$

where *m* is the mass of active material placed on a single electrode, *j* is the constant current, ∫*V* d*t* is the area under the discharge curve, *V* is the maximum operating voltage, and *V_i_* and *V*_f_ are the initial and final voltage values.^10,45,51,52^ Moreover, the equivalent series resistance (ESR) and internal resistance for both cells (SC and SC@KI) have been evaluated by measuring the voltage drop (Δ*V*) upon the current reversal (*j*), from the discharge curve, by using the following expression.^10,44,45^

9 $$\mathrm{ESR}=\frac{\Delta V}{2j}$$

The values of ESR and specific capacitance, i.e., *C*_sp_, from the discharge curve have been calculated for both cells (SC and SC@KI) and listed in Table 2. The SC@KI cell shows almost 5 times higher *C*_sp_ values with respect to SC, due to the dominant interfacial pseudocapacitive effect. In addition to that, ESR is nearly halved, directly affecting the performance of the supercapacitor, as listed in Table 2. The lower value of ESR is associated with the improved ionic conductivity and charge transfer kinetics, which reduces overall internal resistance compared to the gel electrolyte film with zero amount of KI.

**Table 2:** Parameters Evaluated from GCD Analysis for Both Supercapacitors at a Constant Current Density of 1 A g^–1^

EDLCs	ESR (Ω cm^2^)	*C*_sp_ (F g^–1^)	*E*_sp_ (Wh kg^–1^)	*P*_eff_ (W kg^–1^)
SC	23.5 ± 1.5	83 ± 3	6 ± 1	325 ± 5
SC@KI	9 ± 1	426 ± 11	33.5 ± 0.5	232.5 ± 2.5

SC and SC@KI cells have also been charged–discharged at varying current densities in the 1–10 A g^–1^ range to analyze the rate capability of the supercapacitors, as represented in Figures 6C and S7 for SC@KI and SC cells, respectively. The SC@KI cell shows nonlinear GCD patterns due to pseudocapacitive effects, while the SC cell shows linear GCD curves for all current densities. This indicates that the SC cell has a capacitive nature due to the electrostatic charge storage mechanism, while the SC@KI cell shows superior performance by having an electrostatic charge storage mechanism as well as fast pseudocapacitive reactions. Hence, this indicates the moderate rate capability of the cells (SC and SC@KI).

The *C*_sp_ values have been calculated (eq 7) for each current density (1–10 A g^–1^) and plotted with respect to current density, as indicated in Figure 6D. The SC@KI cell shows almost 4–5 times higher *C*_sp_ as compared to the SC cell for the entire range of current densities (1–10 A g^–1^). This directly indicates the superiority of the SC@KI cell over the SC cell for a wide range of current densities. Moreover, the dominance of pseudocapacitive reactions due to the redox additive along with the electrostatic charge storage mechanism can be more clearly observed in the bar diagram represented in Figure 7A. It should be underlined that the size of the ions in the redox additives also directly affects the performance of supercapacitors because of the fast pseudocapacitive reactions across the interface. Generally, the pore sizes in activated carbons with micropores and mesopores are in the range between <2 nm and 2–50 nm, respectively, which is sufficient to lodge redox additive species iodide (I^–^) and iodide-derived species such as I_3_^–^ and oxidized iodide IO_3_^–^.^11^ The typical sizes of the redox additive species I^–^, I_3_^–^, and oxidized iodide IO_3_^–^ are 0.39 nm, 0.63 nm, and 0.57 nm, respectively.^10,11,48^ These ionic redox species have a small enough size for fast, facile switching movements at the interface. Furthermore, they can be easily inserted and deinserted through the pores of the carbon electrodes for fast ion adsorption–desorption. This implies that even at higher scan rates, they can store a significant number of charges for pseudocapacitive and electrostatic charge storage mechanisms, as can be observed in Figures 6D or 7A, where the SC@KI cell shows almost 4–5 times higher *C*_sp_ as compared to the SC cell for the entire range of current densities.

**Figure 7:**
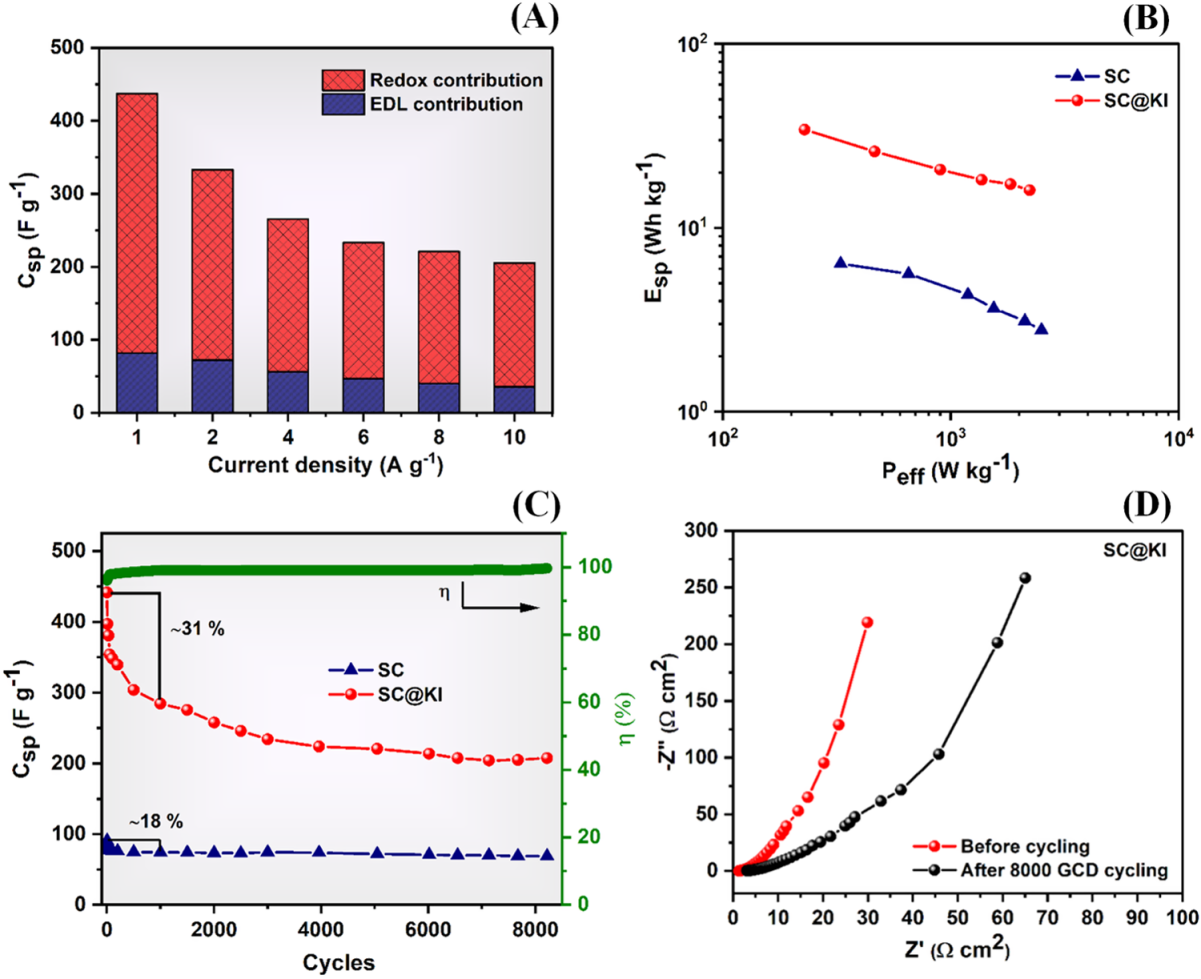
(A) Specific capacitance bar plot with pseudocapacitive and electrostatic charge storage contribution, (B) comparative Ragone plot for both cells (SC and SC@KI), (C) Coulombic efficiency and specific capacitance versus cycle number plot recorded at a current density of 1 A g^–1^, and (D) EIS spectra recorded before GCD cycling and after 8000 cycles for the SC@KI cell.

Specific energy and power have been evaluated from the following expressions^10,11^

10 $$E_{\mathrm{sp}}=\frac{j\times \int Vdt}{2m}$$

11 $$P_{\mathrm{eff}}=\frac{E_{\mathrm{sp}}}{{\Delta t}_{\mathrm{dis}}}$$

where *j* is the constant current, *m* is the mass of the active material coated on a single electrode, ∫*V* d*t* is the area under the discharge curve, and Δ*t*_dis_ is the discharge time.^10,44–46^ Both parameters have been evaluated at 1 A g^–1^ for each cell and are listed in Table 2.

As listed in Table 2, the SC@KI cell shows almost a 5 times increase in specific energy as compared to the SC cell, which is directly associated with the enhanced value of *C*_sp_ (discussed above) due to pseudocapacitive effects. Slightly lower values of specific power have been observed for SC@KI with respect to SC due to the redox additive, which affects the speed of the charge storage mechanism at the interface. However, the specific powers of SC@KI and SC are still comparable. Specific energy and power of supercapacitor cells SC and SC@KI are compared in the Ragone plot shown in Figure 7B. Both cells show the typical nature of supercapacitor cells, an increase in energy at the expense of power.^1,2,44,46^ The direct effect of the pseudocapacitive contributions can be seen in the SC@KI cell, as an increase in specific energy is followed by a decrease in specific power. The optimized value of specific energy has been found to be ∼34 Wh kg^–1^ at a specific power of ∼ 235 W kg^–1^ for the redox additive-based cell (SC@KI). This optimized value of specific energy has been found to be substantially greater or comparable to many recent reported works for supercapacitors based on redox-additive quasi-solid-state electrolytes. In Table 3, the performance of SC@KI is compared to recently reported similar devices.

**Table 3:** Redox-Additive Electrolytes (Liquid/Gel Electrolytes)-Based Carbon Supercapacitors: Comparative Analysis

S. no.	electrode material	electrolyte	redox additive	specific capacitance (F g^–1^)	energy density (Wh kg^–1^)	ref
(a) Liquid Electrolytes with Redox Additive-Based Supercapacitors						
1	rGO	KOH	KI	500	44	^53^
2	AC	H_2_SO_4_	KI	912	19.04	^48^
3	porous carbon	EMITFSI	EMII	200.6	175	^54^
4	porous carbon	EMIBF_4_	EMII	245	36	^55^
5	AC	LiClO_4_/AN	PPD	68.6	54.5	^56^
6	AC	Et_3_NHTFSI	Et_3_NHI	41	10	^57^
7	AC	PYR_14_TFSI	*p*-BQ	156	30	^4^
8	activated charcoal	Et_3_NHTFSI	HQ	72	31.2	^58^
(b) Gel Electrolytes with Redox Additive-based Supercapacitors						
9	AC	PVA + KOH	KI	236.9	7.8	^59^
10	rGO	PVA + KOH	KI	298		^53^
11	AC	PVA + Li_2_SO_4_	BMII	384	29.3	^60^
12	AC	PVdF-HFP + BMITFSI	NaI	334	26.1	^61^
13	AC	PVdF-HFP + BMITFSI	KI	211	46	^10^
14	AC	PVdF-HFP + EMITFSI	KI	250	65	^44^
15	AC	PVAPB	KI	164.7	14.38	^62^
16	AC	pectin + LiCl	KI	437	34	this work

Furthermore, the charge–discharge reversibility of supercapacitors (SC and SC@KI) has been tested by performing 8000 cycles. Their specific capacitance (*C*_sp_) and Coulombic efficiency η (%) have been calculated and plotted against cycle number, as shown in Figure 7C. The Coulombic efficiency η (%) of the cells (SC and SC@KI) has been evaluated from the following expression

12 $$\eta =\frac{t_{D}}{t_{C}}\times 100\%$$

where *t*_D_ and *t*_C_ are the discharging and charging times at constant current, respectively. The redox additive-based supercapacitor SC@KI shows ∼31% fading in *C*_sp_ value for the initial 1000 cycles. After that, a gradual decrease has been observed up to 8000 cycles. Throughout the entire range of cyclic measurements, the SC@KI cell shows significantly higher specific capacitance values thanks to SC, and after 8000 cycles, it is still able to deliver almost double the capacitance as compared to the SC cell. This shows the effectiveness of pseudocapacitive reactions at the interface, which are stable and highly reversible, also for a large number of charge–discharge cycles. Moreover, the SC cell shows almost 18% fading in *C*_sp_ after 1000 cycles, as shown in Figure 7C. This initial fading in specific capacitance is the characteristic of activated carbon-based supercapacitors^44,46,48^ and is caused by irreversible reactions between ions of the electrolyte and/or redox species with the surface functional group on the surface of activated carbon materials.^10^ In addition to that, another reason for this initial sharp fading is the small pore/micropore blockage during initial charge–discharge cycling.^10^ This initial fade could be resolved by implementing a mild electrochemical preconditioning protocol such as low-current cycling for a limited number of initial cycles, which could enhance the electrolyte/electrode interface stabilization and reduce early-stage degradation. Moreover, the cells exhibit almost 100% Coulombic efficiency during the GCD cycling after an initial sharp increase in η (%), as shown in Figure 7C. This further implies the stable charge–discharge performance of cells (SC and SC@KI). Furthermore, the EIS spectra of the SC@KI cell after 8000 GCD cycling were also recorded and compared with the initial spectra, as shown in Figure 7D. The capacitance fading can be attributed to higher resistive values including both charge transfer and ion diffusion resistance after prolonged cycling, which indicates that it undergoes electrochemical and possibly mechanical degradation over long cycling, affecting interfacial stability and ionic transport.

The mechanical stability and flexibility of the SC@KI cell were evaluated by performing bending tests at different angles^63–65^ (0, ∼40°, and ∼80°), as shown in Figure 8A. The prepared cell was subjected to bending while under GCD measurements to assess its performance under mechanical deformation. Figure 8B shows the GCD curves recorded at a current density of 1.3 A g^–1^. The prepared supercapacitor maintained a nearly triangular GCD pattern under all conditions, which is characteristic of ideal capacitive behavior. However, a slight decrease in discharge time is observed with increasing bending angle, which is possibly due to the temporary changes in internal contact resistance due to strain at the electrode/electrolyte interface.

**Figure 8:**
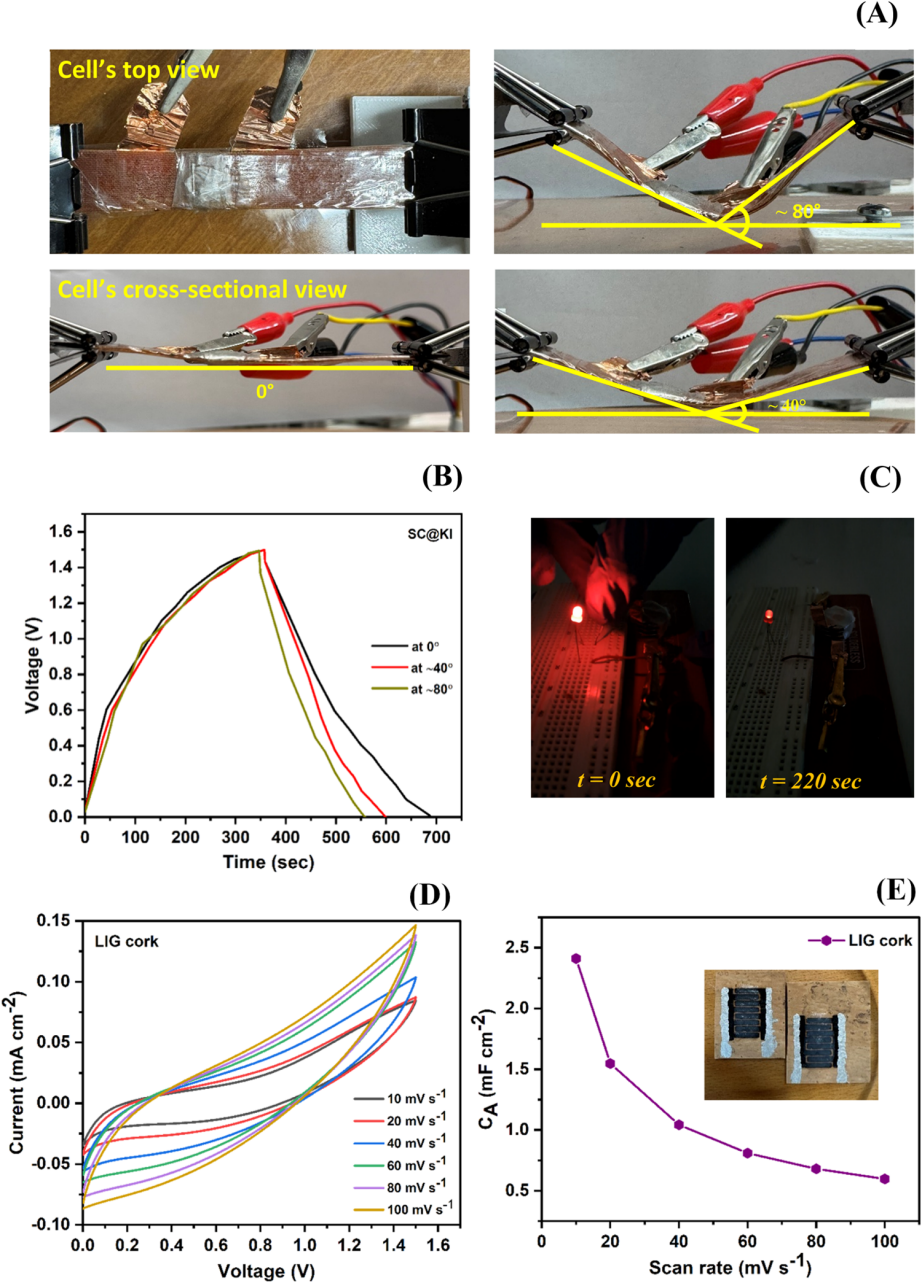
(A) Photographs of supercapacitors (SC@KI) taken at different bending angles. (B) GCD curves obtained at different bending angles recorded at a current density of 1.3 A g^–1^. (C) Images of an SC@KI-powered LED. (D and E) CV and *C*_sp_ versus scan rate plot of the cork-based LIG supercapacitor.

The practical applicability of the prepared redox additive-based supercapacitor (SC@KI) has been tested by successfully powering an LED, as shown in Figure 8C. Three optimized SC@KI cells were connected in series and charged up to ∼4.5 V, powering the LED for about ∼4 min, as can be seen in Figure 8C. This shows a substantial amount of energy storage in the SC@KI cell. Additionally, the potential of the prepared redox additive-based gel electrolyte (GPE-KI) for other applications has been tested by fabricating a cork-based laser-induced graphene (LIG cork) supercapacitor. The liquid solution of GPE-KI was directly cast on the LIG cork and dried overnight at ∼40–45 °C. After that, they were tested by CV analysis, as represented in Figure 8D,E. The photograph of the prepared supercapacitor is shown in the inset of Figure 8E. The maximum specific areal capacitance was ∼2.4 mF cm^–2^ at 10 mV s^–1^, which is higher than the recent reported literature on cork-based supercapacitors.^3^ This shows the potential for other applications of the prepared redox additive-based gel electrolyte film.

## 4 Conclusions

In this work, we successfully synthesized gel electrolyte films by using natural biodegradable polymer pectin, LiCl, and redox additive agent KI for high-performance activated carbon-based supercapacitors. Gel electrolyte films with and without redox additives were prepared for comparative studies. Pectin, being a natural polymer, does not have the same kind of ion transport mechanism as synthetic polymers such as PVdF-HFP or PVA, due to its hydrophilic and rigid structure. For this reason, the redox additive (KI) was added to the gel electrolyte film to increase the ionic conductivity of the gel electrolyte and enhance the pseudocapacitive reactions at the electrolyte–electrode interface. Moreover, introducing a redox additive (KI) to the films increases the mechanical flexibility and thermal stability of the gel electrolyte films. The optimized redox-additive gel electrolyte film (GPE-KI) offers high ionic conductivity, i.e., ∼4.3 × 10^–2^ S cm^–1^, at room temperature and a wide electrochemical voltage window (∼2 V vs Ag/Ag^+^). Symmetric carbon electrode (derived from melon waste)-based supercapacitors were fabricated with redox additive and without redox additive gel electrolytes (GPE-KI and GPE, respectively). The prepared supercapacitors were electrochemically tested by EIS, CV, and GCD measurements. The performance of the redox-additive gel electrolyte-based supercapacitor (SC@KI) improved substantially, with specific capacitance and energy increasing by 4–5 times, from ∼85 to ∼437 F g^–1^ and from ∼7 to ∼34 Wh kg^–1^, respectively, which are favorably comparable to most of the recent reported literature values. Furthermore, the optimized cell (SC@KI) shows moderate rate capability and excellent cycling stability with 99–100% Coulombic efficiency up to ∼8000 cycles. Hence, the introduction of redox additives in gel electrolyte films is an effective approach to enhance the performance of supercapacitors, and this approach is very effective in utilizing the natural biodegradable polymer-based gel electrolytes for designing super high-performance supercapacitors with a very low environmental impact.

## Supplementary Material



## Notes

Photographs of prepared GPEs (Figure S1); SEM images of pectin and GPE films at various magnifications (Figure S2); fitted parameters of supercapacitor cells from the equivalent circuit (Table S3); and electrochemical analysis data including CV and GCD for the SC cell (Figures S4–S7) (PDF)

## References

[1] Ahmad N., Rinaldi A., Sidoli M., Magnani G., Vezzoni V., Scaravonati S., Pasetti L., Fornasini L., Gupta H., Tamagnone M., Ridi F., Milanese C., Riccò M., Pontiroli D. (2024). Pre-Treated Biomass Waste Melon Peels
for High Energy Density Semi Solid-State Supercapacitors. J. Power Sources.

[2] Pontiroli D., Scaravonati S., Magnani G., Fornasini L., Bersani D., Bertoni G., Milanese C., Girella A., Ridi F., Verucchi R., Mantovani L., Malcevschi A., Riccò M. (2019). Super-Activated
Biochar from Poultry
Litter for High-Performance Supercapacitors. Microporous Mesoporous Mater..

[3] Imbrogno A., Islam J., Santillo C., Castaldo R., Sygellou L., Larrigy C., Murray R., Vaughan E., Hoque M. K., Quinn A. J., Iacopino D. (2022). Laser-Induced
Graphene Supercapacitors
by Direct Laser Writing of Cork Natural Substrates. ACS Appl. Electron. Mater..

[4] Navalpotro P., Palma J., Anderson M., Marcilla R. (2016). High Performance Hybrid
Supercapacitors by Using Para-Benzoquinone Ionic Liquid Redox Electrolyte. J. Power Sources.

[5] Zhang L., Yang S., Chang J., Zhao D., Wang J., Yang C., Cao B. (2020). A Review of
Redox Electrolytes for
Supercapacitors. Front. Chem..

[6] Béguin F., Frackowiak E. (2013). Supercapacitors: Materials, Systems,
and Applications. Supercapacitors.

[7] Boyea J. M., Camacho R. E., Sturano S. P., Ready W. J. (2007). Carbon Nanotube-Based
Supercapacitors: Technologies and Markets. Nanotechnol.
Law Bus..

[8] Xu Y., Ren B., Wang S., Zhang L., Liu Z. (2018). Carbon Aerogel-Based
Supercapacitors Modified by Hummers Oxidation Method. J. Colloid Interface Sci..

[9] Mohit, Hashmi S. A. (2023). Biodegradable Poly-ε-Caprolactone
Based Porous Polymer Electrolytes for High Performance Supercapacitors
with Carbon Electrodes. J. Power Sources.

[10] Yadav N., Hashmi S. A. (2020). Energy Enhancement
of Quasi-Solid-State Supercapacitors
Based on a Non-Aqueous Gel Polymer Electrolyte via a Synergistic Effect
of Dual Redox Additives Diphenylamine and Potassium Iodide. J. Mater. Chem. A.

[11] Hor A. A., Yadav N., Hashmi S. A. (2023). Enhanced
Energy Density Quasi-Solid-State
Supercapacitor Based on an Ionic Liquid Incorporated Aqueous Gel Polymer
Electrolyte with a Redox-Additive Trimethyl Sulfoxonium Iodide. J. Energy Storage.

[12] Zhong C., Deng Y., Hu W., Qiao J., Zhang L., Zhang J. (2015). A Review of Electrolyte Materials
and Compositions for Electrochemical
Supercapacitors. Chem. Soc. Rev..

[13] Brandt A., Isken P., Lex-Balducci A., Balducci A. (2012). Adiponitrile-Based
Electrochemical Double Layer Capacitor. J. Power
Sources.

[14] Polymer Electrolytes: Fundamentals and Applications—Google Books, 2024. https://books.google.it/books?hl=en&lr=&id=vHl0AgAAQBAJ&oi=fnd&pg=PP1&ots=72gDEO1Wj8&sig=XJiFYAdsjK4Zfpn-rQwptrF7dDg&redir_esc=y#v=onepage&q&f=false.

[15] Gunday S. T., Cevik E., Bozkurt A., Iqbal A., Asiri S., AlGhamdi A., Almofleh A., Qahtan T. F., Al-Fares F. A. Y., Isık O. (2023). Nonflammable
Supramolecular Polymer Electrolytes for
Flexible and High-Performance Supercapacitor Applications. Energy Fuels.

[16] Gunday S. T., Cevik E., Asiri S., Iqbal A., Almofleh A., Alqarni A. N., Anil I., Alagha O., Bozkurt A. (2022). Synthesis
of Boron-Doped Non-Flammable Anhydrous Electrolytes for Flexible Quasi-Solid-State
Supercapacitor Applications. Energy Fuels.

[17] Sengupta S., Kundu M. (2024). High-Performance Flexible
Planner Device Featuring WS2 Electrode
and Non-Aqueous Polymeric Ionic Electrolytes Incorporated with Ionic
Liquid and Dual Redox Additives. Chem. Eng.
J..

[18] Sengupta S., Kundu M. (2024). All-Solid-State Flexible
Asymmetric Supercapacitors Based on WS2/RGO/CNT
Hybrid Electrodes and Polymer-Based Ionic Liquid Electrolytes. ACS Appl. Energy Mater..

[19] Hor A. A., Ahmad N., Hashmi S. A. (2025). Hierarchical Porous Carbon Electrodes
from Fishtail-Palm Tree Barks for High Performance Quasi-Solid-State
Supercapacitors. J. Energy Storage.

[20] Railanmaa A., Kujala M., Keskinen J., Kololuoma T., Lupo D. (2019). Highly Flexible and Non-Toxic Natural
Polymer Gel Electrolyte for
Printed Supercapacitors for IoT. Appl. Phys.
A: Mater. Sci. Process..

[21] Yadav N., Singh M. K., Yadav N., Hashmi S. A. (2018). High Performance
Quasi-Solid-State Supercapacitors with Peanut-Shell-Derived Porous
Carbon. J. Power Sources.

[22] Jamil R., Silvester D. S. (2022). Ionic Liquid
Gel Polymer Electrolytes for Flexible
Supercapacitors: Challenges and Prospects. Curr.
Opin. Electrochem..

[23] Polymer Electrolytes: Characterization Techniques and Energy Applications - Google Books, 2024. https://books.google.it/books?hl=en&lr=&id=jKm8DwAAQBAJ&oi=fnd&pg=PP2&ots=cIVEEviC-_&sig=vl7DA8m8CZkkqiXERaG47mk2_a8&redir_esc=y#v=onepage&q&f=false.

[24] Liu T., Ren X., Zhang J., Liu J., Ou R., Guo C., Yu X., Wang Q., Liu Z. (2020). Highly Compressible Lignin Hydrogel
Electrolytes via Double-Crosslinked Strategy for Superior Foldable
Supercapacitors. J. Power Sources.

[25] Bai Y., Zhao W., Bi S., Liu S., Huang W., Zhao Q. (2021). Preparation and Application of Cellulose
Gel in Flexible Supercapacitors. J. Energy Storage.

[26] Klobukoski V., Riegel-Vidotti I. C., Vidotti M. (2023). Characterization of Alginate Hydrogel
Electrolytes for Use in Symmetric Supercapacitor Based on Polypyrrole-Modified
Electrodes. Electrochim. Acta.

[27] Yamagata M., Soeda K., Ikebe S., Yamazaki S., Ishikawa M. (2013). Chitosan-Based
Gel Electrolyte Containing an Ionic Liquid for High-Performance Nonaqueous
Supercapacitors. Electrochim. Acta.

[28] Andrade J. R., Raphael E., Pawlicka A. (2009). Plasticized
Pectin-Based Gel Electrolytes. Electrochim.
Acta.

[29] Lersanansit N., Pungjunun K., Chailapakul O., Praphairaksit N. (2024). Development
of Pectin-Based Gel Electrolyte for Wireless Electrochemical Determination
of Cadmium and Lead Using Smartphone. Talanta.

[30] Singhal S., Swami Hulle N. R. (2022). Citrus
Pectins: Structural Properties, Extraction Methods,
Modifications and Applications in Food Systems—A Review. Appl. Food Res..

[31] Chelfouh N., Coquil G., Rousselot S., Foran G., Briqueleur E., Shoghi F., Caradant L., Dollé M. (2022). Apple Pectin-Based
Hydrogel Electrolyte for Energy Storage Applications. ACS Sustainable Chem. Eng..

[32] Xu T., Liu K., Sheng N., Zhang M., Liu W., Liu H., Dai L., Zhang X., Si C., Du H., Zhang K. (2022). Biopolymer-Based
Hydrogel Electrolytes for Advanced Energy Storage/Conversion Devices:
Properties, Applications, and Perspectives. Energy Storage Mater..

[33] Hashmi S. A., Yasir Bhat M., Yadav N. (2020). Gel Polymer Electrolyte
Composition
Incorporating Adiponitrile as a Solvent for High-Performance Electrical
Double-Layer Capacitor. ACS Appl. Energy Mater..

[34] Parveen S., Sehrawat P., Hashmi S. A. (2022). Triglyme-Based
Solvate Ionic Liquid
Gelled in a Polymer: A Novel Electrolyte Composition for Sodium Ion
Battery. Mater. Today Commun..

[35] Yadav N., Yadav N., Hashmi S. A. (2020). Ionic Liquid
Incorporated, Redox-Active
Blend Polymer Electrolyte for High Energy Density Quasi-Solid-State
Carbon Supercapacitor. J. Power Sources.

[36] Lee J., Srimuk P., Fleischmann S., Su X., Hatton T. A., Presser V. (2019). Redox-Electrolytes for Non-Flow Electrochemical
Energy
Storage: A Critical Review and Best Practice. Prog. Mater. Sci..

[37] Sowmiya K. C., Vijayalakshmi K. A. (2024). High Porous Activated Carbon Electrode
Derived from
Watermelon Peel Biomass Exposed with DC Glow Discharge Plasma Applied
for Super Capacitors. ECS J. Solid State Sci.
Technol..

[38] Feng W., Zhang J., Yusuf A., Ao X., Shi D., Etacheri V., Wang D. Y. (2022). Quasi-Solid-State
Sodium-Ion Hybrid
Capacitors Enabled by UiO-66@PVDF-HFP Multifunctional Separators:
Selective Charge Transfer and High Fire Safety. Chem. Eng. J..

[39] Minhas M. U., Ahmad M., Anwar J., Khan S. (2018). Synthesis
and Characterization
of Biodegradable Hydrogels for Oral Delivery of 5-Fluorouracil Targeted
to Colon: Screening with Preliminary In Vivo Studies. Adv. Polym. Technol..

[40] Kiruthika S., Malathi M., Selvasekarapandian S., Tamilarasan K., Moniha V., Manjuladevi R. (2019). Eco-Friendly
Biopolymer Electrolyte,
Pectin with Magnesium Nitrate Salt, for Application in Electrochemical
Devices. J. Solid State Electrochem..

[41] Cevik E., Gunday S. T., Bozkurt A., Iqbal A., Asiri S. M., Alqarni A. N., Almofleh A. (2022). Scalable,
Quasi-Solid-State Bio-Polymer
Hydrogel Electrolytes for High-Performance Supercapacitor Applications. ACS Sustainable Chem. Eng..

[42] Hao X., Zhu J., Jiang X., Wu H., Qiao J., Sun W., Wang Z., Sun K. (2016). Ultrastrong
Polyoxyzole Nanofiber
Membranes for Dendrite-Proof and Heat-Resistant Battery Separators. Nano Lett..

[43] Warburg impedance—PalmSens, 2024. https://www.palmsens.com/knowledgebase-topic/warburg-impedance/.

[44] Hor A. A., Yadav N., Hashmi S. A. (2022). High Energy
Density Carbon Supercapacitor
with Ionic Liquid-Based Gel Polymer Electrolyte: Role of Redox-Additive
Potassium Iodide. J. Energy Storage.

[45] Ahmad N., Rinaldi A., Sidoli M., Magnani G., Morenghi A., Scaravonati S., Vezzoni V., Pasetti L., Fornasini L., Ridi F., Milanese C., Riccò M., Pontiroli D. (2024). High Performance Quasi-Solid-State Supercapacitor Based
on Activated Carbon Derived from Asparagus Waste. J. Energy Storage.

[46] Hor A. A., Hashmi S. A. (2020). Optimization of Hierarchical Porous
Carbon Derived
from a Biomass Pollen-Cone as High-Performance Electrodes for Supercapacitors. Electrochim. Acta.

[47] Taberna P. L., Simon P., Fauvarque J. F. (2003). Electrochemical
Characteristics and
Impedance Spectroscopy Studies of Carbon-Carbon Supercapacitors. J. Electrochem. Soc..

[48] Senthilkumar S. T., Selvan R. K., Lee Y. S., Melo J. S. (2013). Electric Double
Layer Capacitor and Its Improved Specific Capacitance Using Redox
Additive Electrolyte. J. Mater. Chem. A.

[49] Zhang Y. S., Lu C., Hu Y. X., Zhang B. M., Li J., Tian C. Y., Zhang D. T., Kong L. Bin., Liu M. C. (2020). Assemble from 0D
to 3D: Anchored 0D Molybdenum Carbide on 3D Octahedral Amorphous Carbon
with Excellent Capacitive Properties. J. Mater.
Sci..

[50] Jiang J., Zhang Y., An Y., Wu L., Zhu Q., Dou H., Zhang X., Jiang J. M., Zhang Y. D., An Y. F., Wu L. Y., Zhu Q., Dou H., Zhang X. G. (2019). Engineering
Ultrathin MoS2 Nanosheets Anchored on N-Doped Carbon Microspheres
with Pseudocapacitive Properties for High-Performance Lithium-Ion
Capacitors. Small Methods.

[51] Ahmed S., Ahmed A., Rafat M. (2018). Supercapacitor Performance
of Activated
Carbon Derived from Rotten Carrot in Aqueous, Organic and Ionic Liquid
Based Electrolytes. J. Saudi Chem. Soc..

[52] Gurten
Inal I. I., Aktas Z. (2020). Enhancing the Performance of Activated
Carbon Based Scalable Supercapacitors by Heat Treatment. Appl. Surf. Sci..

[53] Sankar K. V., Kalai Selvan R. (2015). Improved Electrochemical Performances
of Reduced Graphene
Oxide Based Supercapacitor Using Redox Additive Electrolyte. Carbon.

[54] You D. J., Yin Z., Ahn Y. K., Lee S. H., Yoo J., Kim Y. S. (2017). Redox-Active
Ionic Liquid Electrolyte with Multi Energy Storage Mechanism for High
Energy Density Supercapacitor. RSC Adv..

[55] Tooming T., Thomberg T., Siinor L., Tõnurist K., Jänes A., Lust E. (2014). A Type High Capacitance
Supercapacitor
Based on Mixed Room Temperature Ionic Liquids Containing Specifically
Adsorbed Iodide Anions. J. Electrochem. Soc..

[56] Yu H., Wu J., Fan L., Hao S., Lin J., Huang M. (2014). An Efficient
Redox-Mediated Organic Electrolyte for High-Energy Supercapacitor. J. Power Sources.

[57] Skowron P., Frackowiak E., Béguin F. (2014). The Carbon/Iodide Interface in Protic
Ionic Liquid Medium for Application in Supercapacitors. ECS Trans.

[58] Sathyamoorthi S., Suryanarayanan V., Velayutham D. (2015). Organo-Redox Shuttle Promoted Protic
Ionic Liquid Electrolyte for Supercapacitor. J. Power Sources.

[59] Yu H., Wu J., Fan L., Xu K., Zhong X., Lin Y., Lin J. (2011). Improvement of the
Performance for Quasi-Solid-State Supercapacitor
by Using PVA–KOH–KI Polymer Gel Electrolyte. Electrochim. Acta.

[60] Tu Q. M., Fan L. Q., Pan F., Huang J. L., Gu Y., Lin J. M., Huang M. L., Huang Y. F., Wu J. H. (2018). Design
of a Novel Redox-Active Gel Polymer Electrolyte with a Dual-Role Ionic
Liquid for Flexible Supercapacitors. Electrochim.
Acta.

[61] Yadav N., Yadav N., Singh M. K., Hashmi S. A. (2019). Nonaqueous, Redox-Active
Gel Polymer Electrolyte for High-Performance Supercapacitor. Energy Technol..

[62] Yang C., Li D., Gao H., Liu Q., Zhu J., Wang F., Jiang M. (2020). Constructing High-Energy-Density
Aqueous Supercapacitors with Potassium
Iodide-Doped Electrolytes by a Precharging Method. ACS Appl. Energy Mater..

[63] Cevik E., Bozkurt A. (2021). Redox Active Polymer Metal Chelates for Use in Flexible
Symmetrical Supercapacitors: Cobalt-Containing Poly­(Acrylic Acid)
Polymer Electrolytes. J. Energy Chem..

[64] Shar S. S., Cevik E., Bozkurt A., Yaman C., Almutari Z., Kayed T. S. (2020). Molybdate Incorporated
Poly­(Acrylic Acid) Electrolytes
for Use in Quasi-Solid State Carbon Based Supercapacitors: Redox-Active
Polychelates. Electrochim. Acta.

[65] Cevik E., Bozkurt A. (2020). Design of High-Performance
Flexible Symmetric Supercapacitors
Energized by Redox-Mediated Hydrogels Including Metal-Doped Acidic
Polyelectrolyte. Int. J. Energy Res..

