# Supplementary material for: Redox-Additive Gel Polymer Electrolyte Based on the Biodegradable Polymer Pectin for Supercapacitors with Enhanced Thermal Stability
Source: ACS Appl Energy Mater. 2025 Jun 22;8(13):9391–406. doi: 10.1021/acsaem.5c01039 (PMC12264864; doi:10.1021/acsaem.5c01039)
Supplement: Supplementary file 1 [file ae5c01039_si_001.pdf]

Supporting Information

Redox additive gel polymer electrolyte based on biodegradable polymer pectin for supercapacitors with enhanced thermal stability

Niyaz Ahmad[ab], *Alessia Rinaldi*[ab], *Michele Setti*[ab], *Michele Sidoli*[ab], *Silvio Scaravonati*[ab], *Vincenzo Vezzoni*[ab], *Giacomo Magnani*[ab]*, *Mauro Riccò*[ab], *Chiara Milanese*[c], *Maria-Magdalena Titirici*[de], *Daniele Pontiroli*[ab]

[a]Nanocarbon Laboratory, cIDEA & Department of Mathematical, Physical and Computer Sciences, University of Parma, Parco Area delle Scienze 7/A, 43124, Parma, Italy.

[b]GISEL National Centre of Reference for Electrochemical Energy Storage Systems, INSTM National Interuniversity Consortium of Materials Science and Technology, Via Giusti 9, 50121 Firenze, Italy.

[c]Pavia Hydrogen Lab, Chemistry Department, Physical Chemistry Section.
University of Pavia & C.S.G.I., I-27100 Pavia, Italy

[d]Department of Chemical Engineering, Imperial College London, London SW7 2AZ, UK.

[e]Tohoku University, Advanced Institute for Materials Research, 2 Chome-1-1 Katahira, Aoba Ward, Sendai, Miyagi 980-8577, Japan.

* Corresponding author. Tel. +39 0521 905282. E-mail: giacomo.magnani@unipr.it (Giacomo Magnani)

(S1)

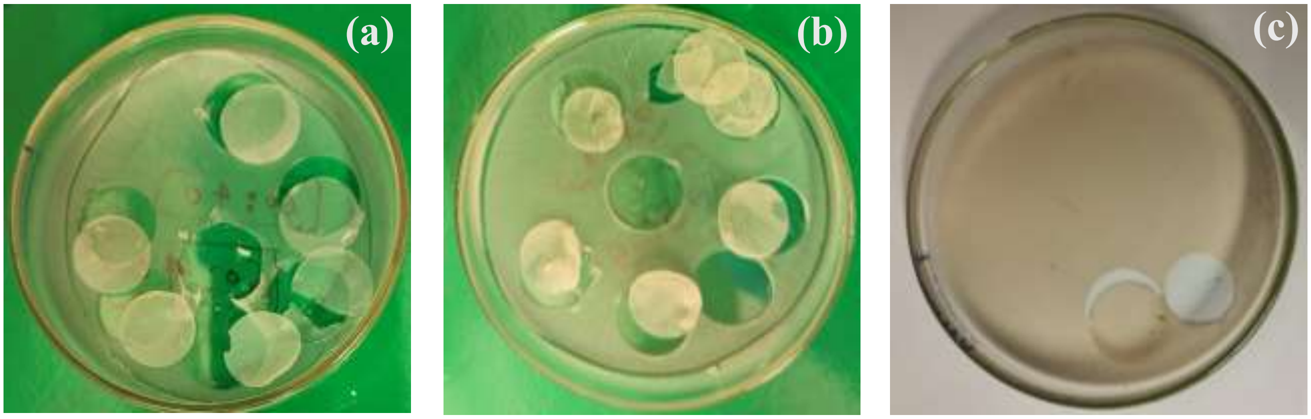

Pectin:LiCl (30:70)

Pectin:LiCl (40:60)

Pectin:LiCl (50:50)

(S2)

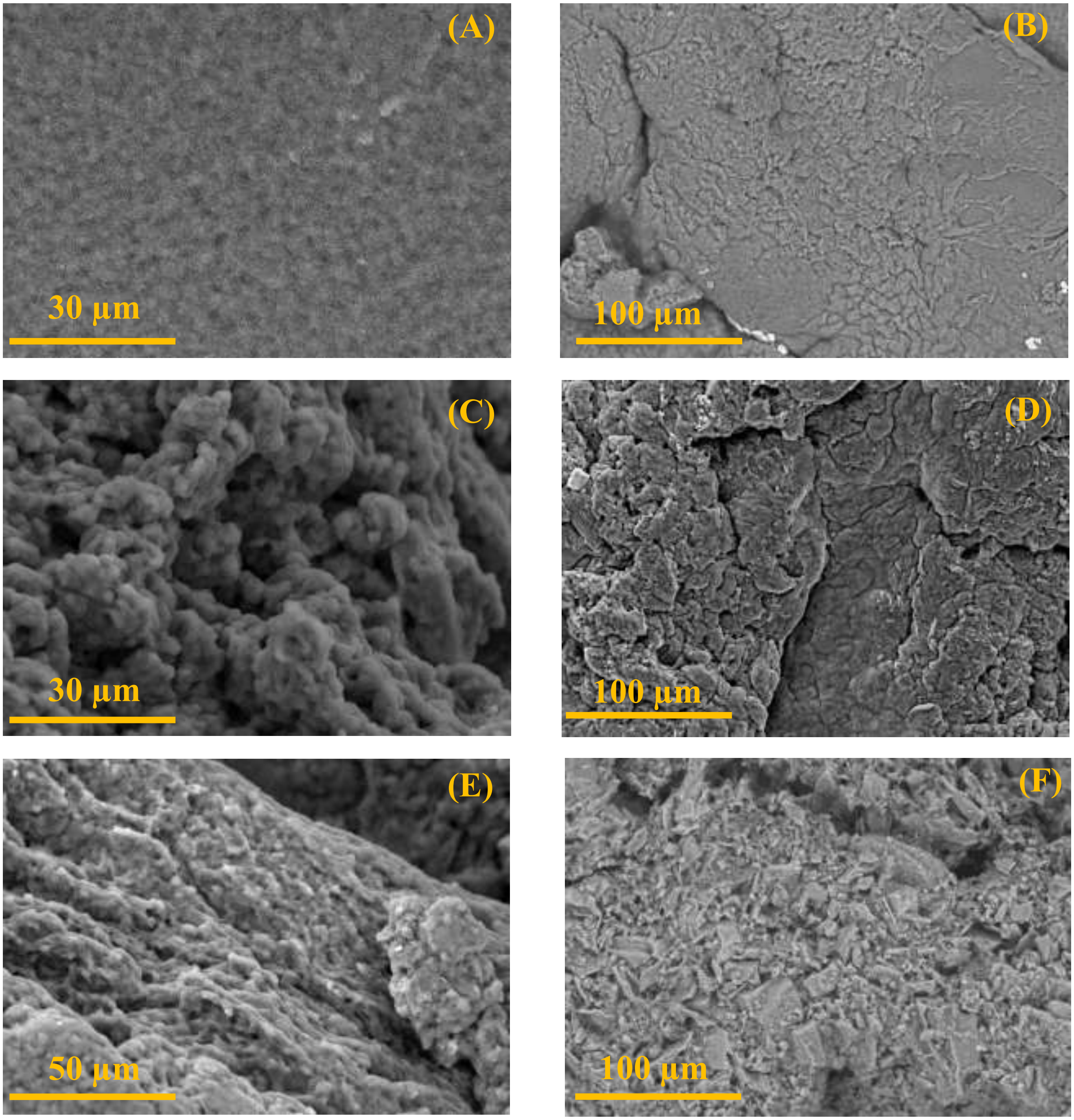

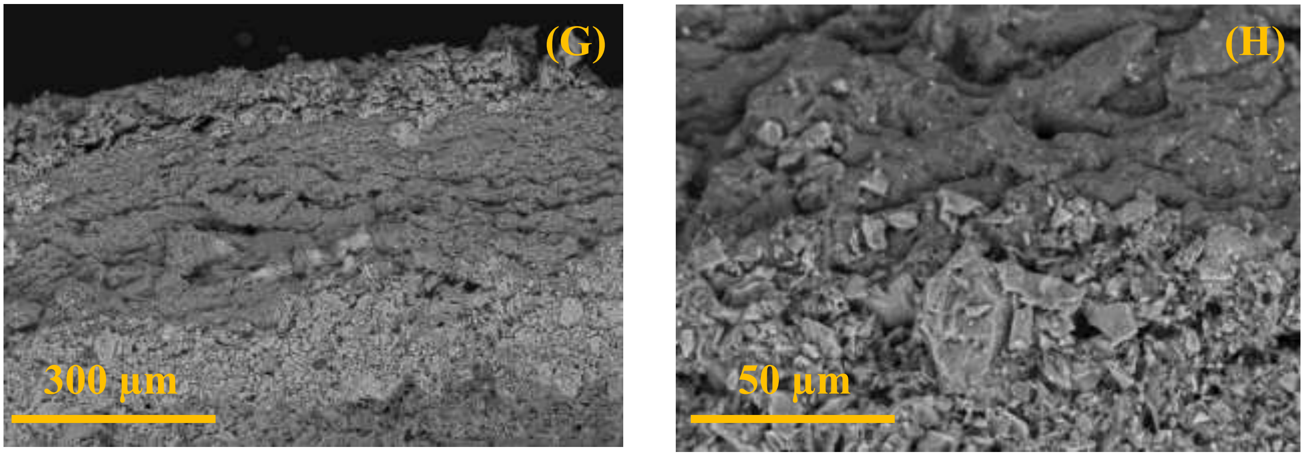

Figure S2: SEM images of films at different magnifications (A) pure pectin at 1500x, (B & C) GPE at 400x and 1500x, respectively, and (D & E) GPE-KI at 400x and 1000x, respectively (F) SEM image of carbon electrode at 500x and (G & H) the interface of the electrode/electrolyte at 150x and 600x, respectively.

Table S3

Fitted parameters of capacitor cells by equivalent circuits.

EDLCs	R_1 / Ω cm^2	R_2 / Ω cm^2	CPE Q (Ω cm^2), n	W (Ω s^{-1} cm^{-2})	W_{o1} W_{or1} (Ω $s^{0.5}$ $cm^{-0.5}$), W_{oc1} (Ω $s^{-0.5}$ $cm^{-0.5}$),
**SC**	6.38	0.31	3.1×10^{-4}, 0.788	16.529	8.1×10^{-18}, 2.54×10^{-3}
**SC@KI**	1.35	0.1305	3.3×10^{-4}, 0.91	8.67	1.4×10^{-8}, 7.1×10^{-10}

(S4)

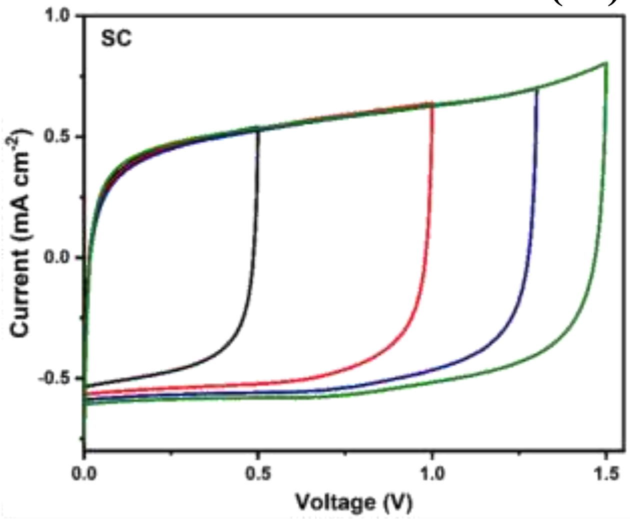

(S5)

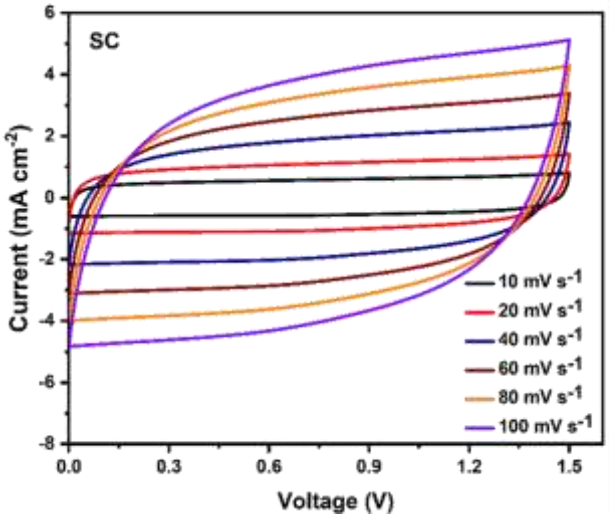

(S6)

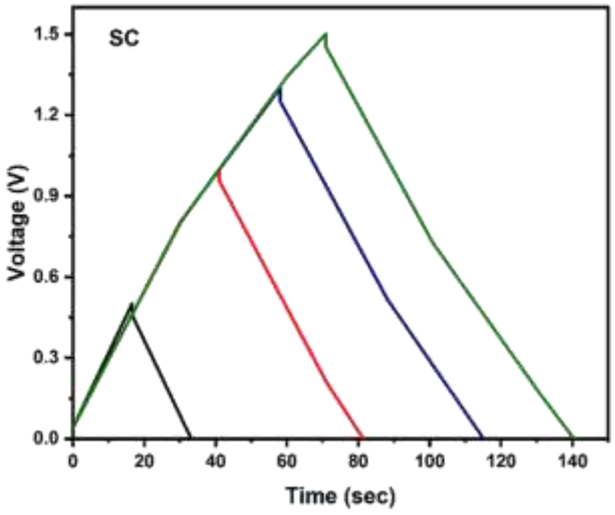

(S7)

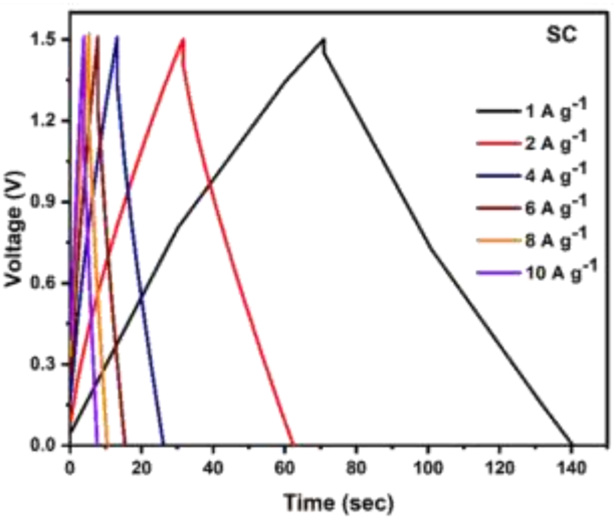

Figure S4: CV patterns for varying voltage ranges from 0.5 V to 1.5 V at a constant scan rate of 10 mV/s for cell SC.

Figure S5: CV patterns for varying scan rates from 10 to 100 mV/s for cell SC.

Figure S6: charge-discharge profiles of cell SC in the varying range of potential 0.5- 1.5 V at 1 A/g.

Figure S7: GCD curves at varying current densities from 1 to 10 A/g in the potential range 0 to 1.5 V for cell SC.
